# The TOR kinase pathway is relevant for nitrogen signaling and antagonism of the mycoparasite *Trichoderma atroviride*

**DOI:** 10.1371/journal.pone.0262180

**Published:** 2021-12-31

**Authors:** Rossana Segreto, Hoda Bazafkan, Julia Millinger, Martina Schenk, Lea Atanasova, Maria Doppler, Christoph Büschl, Mélanie Boeckstaens, Silvia Soto Diaz, Ulrike Schreiner, Fabiano Sillo, Raffaella Balestrini, Rainer Schuhmacher, Susanne Zeilinger

**Affiliations:** 1 Department of Microbiology, University of Innsbruck, Innsbruck, Austria; 2 Institute of Food Technology, University of Natural Resources and Life Sciences (BOKU), Vienna, Austria; 3 Department of Agrobiotechnology IFA-Tulln, Center for Analytical Chemistry, University of Natural, Resources and Life Sciences, Vienna (BOKU), Tulln, Austria; 4 Département de Biologie Moléculaire, Laboratory of Biology of Membrane Transport, Université Libre de Bruxelles, Gosselies, Belgium; 5 IPSP-CNR, Torino, Italy; University of Nebraska-Lincoln, UNITED STATES

## Abstract

*Trichoderma atroviride* (Ascomycota, Sordariomycetes) is a well-known mycoparasite applied for protecting plants against fungal pathogens. Its mycoparasitic activity involves processes shared with plant and human pathogenic fungi such as the production of cell wall degrading enzymes and secondary metabolites and is tightly regulated by environmental cues. In eukaryotes, the conserved Target of Rapamycin (TOR) kinase serves as a central regulator of cellular growth in response to nutrient availability. Here we describe how alteration of the activity of TOR1, the single and essential TOR kinase of *T*. *atroviride*, by treatment with chemical TOR inhibitors or by genetic manipulation of selected TOR pathway components affected various cellular functions. Loss of TSC1 and TSC2, that are negative regulators of TOR complex 1 (TORC1) in mammalian cells, resulted in altered nitrogen source-dependent growth of *T*. *atroviride*, reduced mycoparasitic overgrowth and, in the case of Δ*tsc1*, a diminished production of numerous secondary metabolites. Deletion of the gene encoding the GTPase RHE2, whose mammalian orthologue activates mTORC1, led to rapamycin hypersensitivity and altered secondary metabolism, but had an only minor effect on vegetative growth and mycoparasitic overgrowth. The latter also applied to mutants missing the *npr1-1* gene that encodes a fungus-specific kinase known as TOR target in yeast. Genome-wide transcriptome analysis confirmed TOR1 as a regulatory hub that governs *T*. *atroviride* metabolism and processes associated to ribosome biogenesis, gene expression and translation. In addition, mycoparasitism-relevant genes encoding terpenoid and polyketide synthases, peptidases, glycoside hydrolases, small secreted cysteine-rich proteins, and G protein coupled receptors emerged as TOR1 targets. Our results provide the first in-depth insights into TOR signaling in a fungal mycoparasite and emphasize its importance in the regulation of processes that critically contribute to the antagonistic activity of *T*. *atroviride*.

## Introduction

Several *Trichoderma* species are potent mycoparasites known as biocontrol agents for the protection of plants against fungal phytopathogens. Necrotrophic mycoparasites such as *Trichoderma* kill other fungi through the production of damaging enzymes and toxic metabolites followed by feeding on the released nutrients [1]. While the mechanisms contributing to the mycoparasitic activity are well characterized, several knowledge gaps exist about the regulatory processes and pathways involved in activating the mycoparasitic response. During recent years, evidence accumulated that G protein signaling, the cAMP pathway, and mitogen-activated protein kinase (MAPK) cascades affect growth and development as well as the mycoparasitic activity in response to environmental cues (reviewed in [1, 2]).

The target of rapamycin (TOR) pathway is a central regulator of cell growth in response to nutrient availability in eukaryotes. Higher eukaryotes possess only one evolutionary conserved TOR protein kinase, while some yeast species such as *Saccharomyces cerevisiae* (Ascomycota, Saccharomycotina) possess two, which are active under nitrogen sufficiency and inactivated upon nitrogen starvation or by the addition of the bacterial metabolite rapamycin [3]. In yeast, the rapamycin-triggered TOR inhibition notably causes nuclear accumulation of the GATA-type transcription factor Gln3 resulting in the expression of nitrogen catabolite-repressed genes that are required for the assimilation of alternative nitrogen sources. This leads to the production of transport proteins for nitrogenous compounds, such as the general amino acid permease Gap1p and the ammonium transport proteins Mep1p, Mep2p and Mep3p, whose activity and/or stability are moreover post-translationally regulated by the protein kinase Npr1 (nitrogen permease reactivator 1) [4–7]. Under nutrient-rich conditions, when TOR is active, Npr1 is inhibited by phosphorylation. In case of TOR inhibition by rapamycin, Npr1 becomes functional allowing the TOR pathway to rapidly adjust the permeability of nutrients to regulate cell growth in response to environmental nutrient availability [8–10].

The TOR kinase functions in two distinct protein complexes, TORC1 and TORC2 [11]. Mammalian TORC1 governs metabolism and cell growth by promoting ribosome biogenesis and protein synthesis through triggering the phosphorylation of the S6 subunit of the ribosomal complex (Rps6) and the eukaryotic translation initiation factor 4E binding protein [12]. Similarly, under preferential growth conditions, *S*. *cerevisiae* TORC1 stimulates anabolic processes leading to ribosome and protein biosynthesis by activating the S6-Sch9 signaling branch, whereas it represses the Tap42-PP2A phosphatase branch required for the activation of environmental stress response, amino acid biosynthesis, nitrogen assimilation pathways and the catabolic process of autophagy [13–17]. The functions of TORC2 are less well understood. TORC2 regulates actin polarization and cytoskeleton rearrangement in most organisms studied and, in *S*. *cerevisiae*, also cell wall integrity [18, 19]. Rapamycin is an allosteric inhibitor of mainly TORC1, while TORC2 is largely unaffected. ATP-competitive inhibitors such as Torin1 and Torin2 target the kinase activity and hence act on both TORC1 and TORC2. In addition, they may as well cause inhibition of rapamycin-resistant TORC1 functions such as observed in mammalian and *Schizosaccharomyces pombe* (Ascomycota, Schizosaccharomycetes) cells [20, 21].

Some, but not necessarily all, components of the TOR pathway are present in animals, fungi, plants and protozoa. Complete genome sequence analyses revealed that the pathway evolved by successive addition of extra features from a primitive energy-sensing system in the ancestral eukaryote that coupled cell growth to energy supplies [22]. TSC1 and TSC2, which form the tuberous sclerosis complex (TSC), have been studied mainly in mammalian cells where TSC regulates TORC1 activity via the small G protein Rheb (Ras homolog enriched in brain) in response to various cues including specific nutrients and growth factors [23]. In addition, TSC is thought to positively regulate mammalian TORC2 independently of Rheb which, however, is poorly understood [18]. A similar situation exists in *S*. *pombe* [24, 25], whereas *S*. *cerevisiae* lacks TSC1/2 homologs but encodes a Rheb-like protein (Rhb1) [26, 27]. *Aspergillus fumigatus* (Ascomycota, Eurotiomycetes) is the only filamentous fungus in which a Rheb orthologue has been functionally characterized [28]. However, similar to all other Eurotiomycetes examined so far, *A*. *fumigatus* lacks Tsc1 [27]. Transcription of the *A*. *fumigatus rheb* orthologue, *rhbA*, was induced in response to nitrogen starvation and the growth of the *rhbA* deletion mutant was impaired on medium containing poor nitrogen sources [28, 29]. In addition, the Δ*rhbA* mutant showed enhanced sensitivity to the TOR inhibitor rapamycin and reduced virulence in a mouse model of invasive aspergillosis [28].

In fungi, the availability and quality of nutrients influence cellular key processes and nitrogen limitation emerged as one of the cues for activating the expression of virulence-related genes in plant pathogens [30, 31]. Although TOR signaling is poorly studied in filamentous fungi, an important role of this pathway in regulating virulence functions in *Fusarium graminearum*, *F*. *oxysporum*, and *F*. *fujikuroi* (all Ascomycota, Sordariomycetes) became apparent [31–33]. Over the years, evidence accumulated that a similar situation may exist in the mycoparasitic fungus *Trichoderma atroviride* (Ascomycota, Sordariomycetes) in which the presence of a living fungal host causes stress resembling nitrogen limitation [34, 35]. Based on this evidence, we aimed to characterize the role of the hitherto unstudied TOR signaling pathway in *T*. *atroviride*. By using both chemical TOR kinase inhibition as well as genetic manipulation of selected TOR pathway components, we found that *T*. *atroviride* possesses a functional TOR pathway that governs important cellular functions including vegetative growth, ribosome biogenesis, and nitrogen utilization, as well as mycoparasitism-related functions such as mycoparasitic overgrowth and secondary metabolite production.

## Results

### The growth of *T*. *atroviride* is sensitive to TOR kinase inhibitors

**Fig 1:**
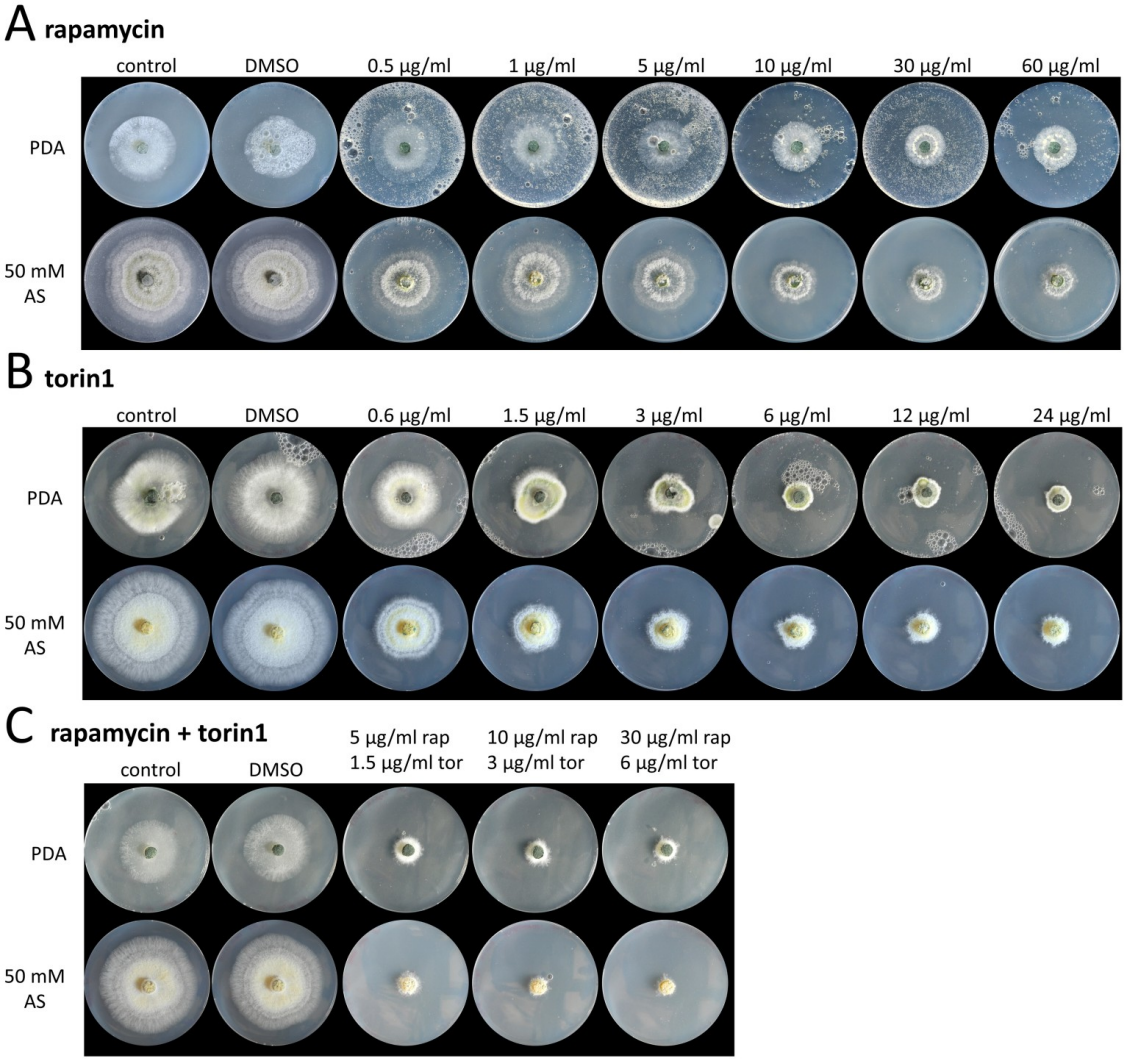
Radial growth of *T*. *atroviride* in the presence of TOR kinase inhibitors. The fungus was cultivated on solid potato dextrose agar (PDA) or minimal medium with 50 mM ammonium sulfate (AS) supplemented with different concentrations of (A) rapamycin, (B) torin1, and (C) different combinations of rapamycin (rap) and torin1 (tor). TOR kinase inhibitors were dissolved in DMSO and respective amounts of DMSO were used as control. Plates were incubated at 25°C for 48 hours in the case of PDA and for 72 hours in the case of minimal medium with 50 mM AS.

The sensitivity to the TOR kinase inhibitor rapamycin was assayed in order to get evidence if TOR signaling governs *T*. *atroviride* growth. Cultivation of *T*. *atroviride* on PDA plates amended with rapamycin revealed inhibition of radial growth at rapamycin concentrations ≥ 10 μg/ml (11 μM). Interestingly, even at higher concentrations (60 μg/ml; 66 μM) rapamycin only inhibited up to 40% of radial growth under the conditions tested (Fig 1A; S1 Table in S1 File). On minimal media (MM) with ammonium sulfate as sole nitrogen source, rapamycin more strongly interfered with fungal growth than on PDA resulting in a slight growth reduction already at a concentration of 500 ng/ml and a ~50% reduced colony size at 10 μg/ml. However, similar to PDA, rapamycin was not able to completely shut-down fungal growth on this medium even at concentrations of up to 60 μg/ml (Fig 1A). When cultivating *T*. *atroviride* on PDA supplemented with the second generation TOR inhibitor torin1, a clear dose-dependent effect on fungal growth (~25 to 80% growth inhibition in the range of 0.6 μg/ml (1 μM)– 6 μg/ml (10 μM) torin1) was observed. Higher concentrations of up to 24 μg/ml (40 μM) did not significantly inhibit growth further (Fig 1B; S1 Table in S1 File). A similar effect emerged on MM. Only combined treatment of the fungus with 30 μg/ml rapamycin and 6 μg/ml torin1 led to a nearly complete (> 90%) growth inhibition on MM (Fig 1C; S1 Table in S1 File). Altogether, the observed effects of the TOR kinase inhibitors suggest the presence of a functional TOR kinase signaling pathway that impacts vegetative growth of *T*. *atroviride*.

### *T*. *atroviride* possesses TORC1 and TORC2 as well as TSC complex components

Based on the evidence of a functional TOR kinase in *T*. *atroviride*, we next aimed to identify and functionally characterize selected components of the TOR pathway in this mycoparasitic fungus. BLASTp analysis of the *T*. *atroviride* genome database with *S*. *cerevisiae* Tor1p and Tor2p [36] revealed a single TOR orthologue (Ta_293155; TOR1) as previously described [37] (S2 Table in S1 File). The predicted *T*. *atroviride* TOR1 protein contains the typical conserved TOR kinase architecture [38] with nine N-terminal HEAT repeats, FAT and FATC domains, a FKBP12-rapamycin binding domain, and the phosphoinositide 3-kinase catalytic domain. Genome analysis also revealed the presence of a rapamycin-binding FKBP12 orthologue (Ta_297179) in *T*. *atroviride*, which is implicated in forming a complex with rapamycin that negatively affects TORC1 activity [39]. Furthermore, orthologues of the yeast TORC1 component Kog1 (Ta_223683), of the TORC2 components Avo1 (Ta_40578), Avo2 (Ta_239629), and Avo3 (Ta_145571) and of Lst8 (Ta_283530), which is an essential component of both TORC1 and TORC2 [40], were found. These data suggest the opportunity for *T*. *atroviride* TOR1 to form both TORC1 and TORC2 complexes.

The Ras-like small GTPase Rheb is one of the major regulators of TORC1 activity in mammalian cells [41]. A single Rheb orthologue, Ta_129536, which we named RHE2, is encoded in the *T*. *atroviride* genome [37] (S2 Table in S1 File). The activity of Rheb is regulated by the tuberous sclerosis complex (TSC), with TSC2 inhibiting TOR signaling by acting as GTPase activator for Rheb [23, 41]. The respective *T*. *atroviride* orthologues of TSC1 and TSC2 were identified as Ta_294527 (TSC1) and Ta_288880 (TSC2). The predicted *T*. *atroviride* TSC1 protein contains a harmatin domain, while the predicted *T*. *atroviride* TSC2 protein harbors a RAP GTPase-activating domain in its C-terminal part being typical for mammalian tuberin [42]. The obtained *in silico* evidence hence indicates the presence of a TSC complex in *T*. *atroviride*, which, based on the conserved GTPase-activating domain in Ta_288880 (TSC2), is supposed to down-regulate RHE2.

**Fig 2:**
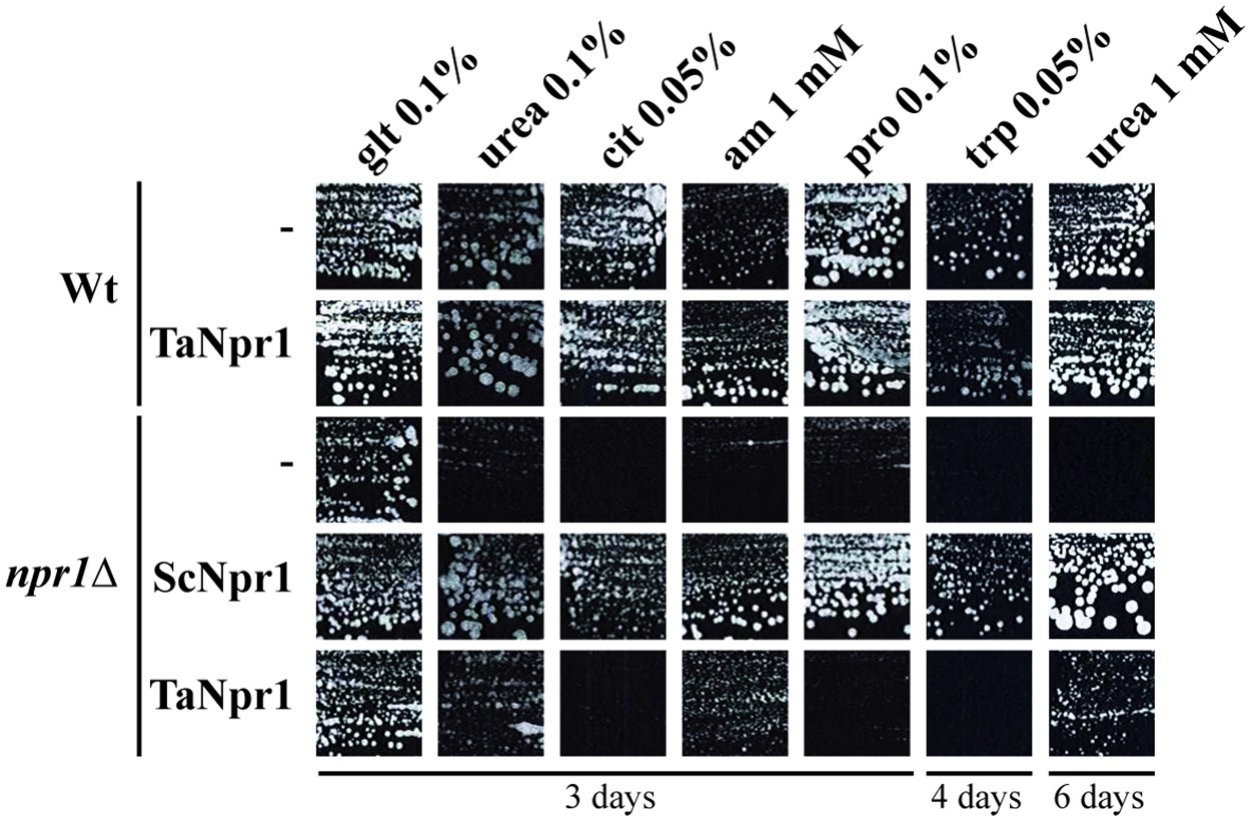
Complementation tests of *S*. *cerevisiae npr1Δ* cells with *T*. *atroviride* NPR1 in the presence of several nitrogen sources. *S*. *cerevisiae* wild type (Wt; *ura3*, 23344c) and *npr1Δ* (*npr1Δ ura3*, PVV357) cells were transformed with the empty plasmid YEplac195 (-), YCpScNpr1 [8] or with YEplac195-TaNpr1p. Cells were grown in minimal buffered (pH 6.1) medium containing 3% glucose as the carbon source and glutamate (glt) 0.1%, ammonium (am) 1 mM, proline (pro) 0.1%, urea 0.1% or 1mM, citrulline (cit) 0.05% or tryptophan (trp) 0.05% as the nitrogen sources. Plates were incubated for 3, 4 or 6 days at 29°C.

Even though a role upstream of TORC1 has been recently suggested for the fungus-specific serine/threonine kinase Npr1, it is overall known as a downstream TORC1 target in *S*. *cerevisiae*, where it is notably involved in the regulation of plasma membrane transporters such as Gap1p and Mep2p [4, 6, 8]. Recently, Pfannmüller et al. [43] reported on three genes encoding Npr1 orthologues in *F*. *fujikuroi* of which *FfNPR1-1* could partially restore the growth defects of the *S*. *cerevisiae npr1*Δ mutant on ammonium, arginine and urea. Querying the *T*. *atroviride* genome database with *S*. *cerevisiae* Npr1p led to Ta_1703 as the by far most significant hit (S2 Table in S1 File). Ta_1703 has 47% amino acid (aa) identities with Npr1p and as well resulted as the best hit when using *F*. *fujikuroi* FfNpr1-1 as query (69% aa identities). With *F*. *fujikuroi* FfNpr1-2 as query, Ta_155476 emerged as the best hit (89% aa identities) while FfNpr1-2 only showed moderate (39%) amino acid identities to Ta_1703. The third Npr1 homologue of *F*. *fujikuroi*, FfNpr1-3, resulted in the identification of an additional *T*. *atroviride* serine/threonine-specific protein kinase (Ta_294017) as the best hit (68% aa identities) followed by Ta_1703 (41% aa identities) and Ta_155476 (38% aa identities). To assess if Ta_1703 (in the following named NPR1-1), which showed the highest similarity to yeast ScNpr1p, is its functional homologue, the full-length cDNA was expressed in *S*. *cerevisiae npr1*Δ mutant cells and yeast growth was tested in the presence of selected nitrogen sources [8]. Growth of yeast *npr1Δ* cells is reduced in the presence of several nitrogen sources, as Npr1 is essential to maintain the activity of different transporters of nitrogenous compounds [5]. As positive control of the growth test, the *npr1*Δ strain expressing *S*. *cerevisiae* Npr1 was used, while *npr1*Δ cells transformed with the empty vector served as negative control. Similar to *F*. *fujikuroi* FfNpr1-1, *T*. *atroviride* NPR1-1 complemented the growth defect of yeast *npr1*Δ cells slightly in the presence of urea and more efficiently in the presence of a low ammonium concentration, but not on the other nitrogen sources tested (Fig 2). These results support the hypothesis established by Pfannmüller et al. [43] that the functions of yeast Npr1 are split among different kinases in *F*. *fujikuroi*, and possibly also in *T*. *atroviride*.

### Interfering with the TOR pathway affects growth and nitrogen source utilization of *T*. *atroviride*

For functional characterization of TOR pathway components in *T*. *atroviride*, we selected *tor1*, *tsc1*, *tsc2*, *rhe2* and *npr1-1* for generating deletion mutants. At least 50 hygromycin-resistant colonies were obtained for each gene. Purification to mitotic stability accompanied by PCR-based screening resulted in six Δ*tsc1*, four Δ*tsc2*, three Δ*rhe2*, and 12 Δ*npr1-1* independent mutants with homologous integration of the deletion cassette at the respective target locus. After having determined the copy number of selected independent strains of the different mutants (S1 Fig) and shown that the strains carrying the same deletion behave similar in growth assays (S2 and S3 Figs), one strain of each mutant was used for further experiments. Attempts to delete the *tor1* gene failed, as the generation of homokaryotic deletion mutants from the obtained hygromycin-resistant colonies that emerged from different rounds of transformation was unsuccessful. Based on these results and previous reports on the lethality of a full disruption of TOR kinase activity in several organisms including fungi [32, 33, 44], we assume that *tor1* is also an essential gene in *T*. *atroviride*.

**Fig 3:**
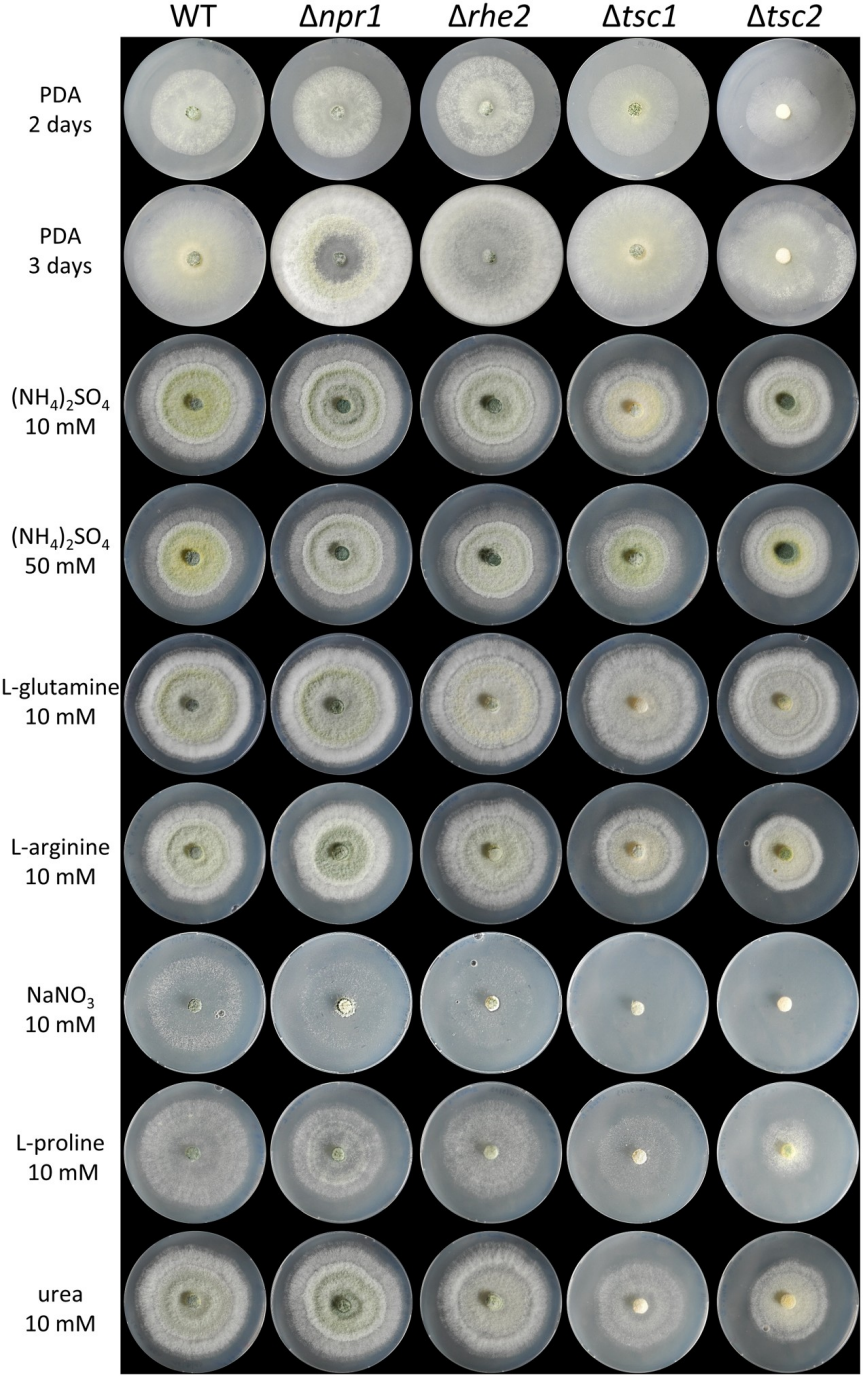
Effect of *npr1*, *rhe2*, *tsc1* and *tsc2* gene deletion on mycelial growth of *T*. *atroviride* on different nitrogen sources. Fungi were grown on PDA or on minimal medium amended with different nitrogen sources (10 or 50 mM ammonium sulfate, 10 mM L-glutamine, 10 mM L-arginine, 10 mM sodium nitrate, 10 mM L-proline, or 10 mM urea) at 25°C for three days. Growth on PDA after two and three days is shown.

Because the TOR pathway is an important regulator of growth, we investigated the effect of the deletion of the selected TOR pathway components on *T*. *atroviride* biomass formation and radial colony growth. Biomass production in liquid complete media (PDB) was decreased in Δ*tsc1* and Δ*tsc2* mutants (by ~25% respectively), whereas Δ*rhe2* and Δ*npr1-1* mutants grew similarly as the wild type (S3 Table in S1 File). Upon cultivation on solid complete media (PDA), a similar picture emerged (Fig 3). The Δ*tsc2* mutant showed decreased radial growth; Δ*tsc1*, however, grew similar to the wild type, while Δ*npr1-1* and Δ*rhe2* mutants showed slightly increased growth. Sporulation was comparable to the wild type in Δ*npr1-1*, Δ*rhe2* and Δ*tsc1* mutants, whereas Δ*tsc2* showed a delay in conidia formation (Fig 3, S2 Fig). The growth behavior of Δ*rhe2* and Δ*npr1-1* mutants on MM supplemented with ammonium sulfate, arginine, or urea was similar to the wild type, while they showed slightly reduced growth on the poor nitrogen sources sodium nitrate and proline. Δ*tsc1* and especially Δ*tsc2* exhibited reduced radial growth on all of the tested nitrogen sources, in particular on nitrate. Interestingly, in the presence of glutamine as sole nitrogen source, all mutants exhibited similar colony sizes as the wild type (Fig 3; S3 Fig).

To get a more global insight into putative alterations in nitrogen utilization resulting from deletion of TOR pathway genes, we performed a comparative phenotypic profiling of the mutants and the wild type on 95 different nitrogenous compounds using the Biolog Phenotype Microarrays. Cluster analysis of the wild type nitrogen source utilization at different time points (24, 48, 72 hours) revealed four distinct clusters and several groups (S4 Fig). Such sister group of clusters I and II contained the dipeptides Gly-Met and Met-Ala which supported the best growth of *T*. *atroviride*. Cluster I also comprised nitrogen sources which yielded high mycelial density. It included mainly L-amino acids such as L-glutamine and L-asparagine, dipeptides, and urea, γ-amino-N-butyric acid and N-acetyl-D-glucosamine, the latter having been described as a preferred carbon source for *T*. *atroviride* as well [45]. Cluster II contained nitrogen sources promoting slower growth of the fungus than those in cluster I. It contained dipeptides and the amino acid L-proline but also ammonium, nitrate, and allantoin, which allowed considerable growth of *T*. *atroviride*. Cluster III nitrogen sources comprised several L- and D-amino acids that were less efficiently catabolized and which promoted only weak growth. Cluster IV nitrogen sources, on which *T*. *atroviride* only marginally grew or was unable to grow, included several amines, the amino sugars N-acetyl-D-mannosamine and N-acetyl-D-galactosamine. Pyrimidines and pyrimidine-derivatives thymine, uracil, thymidine, uridine, and alloxan were also present in cluster IV.

**Fig 4:**
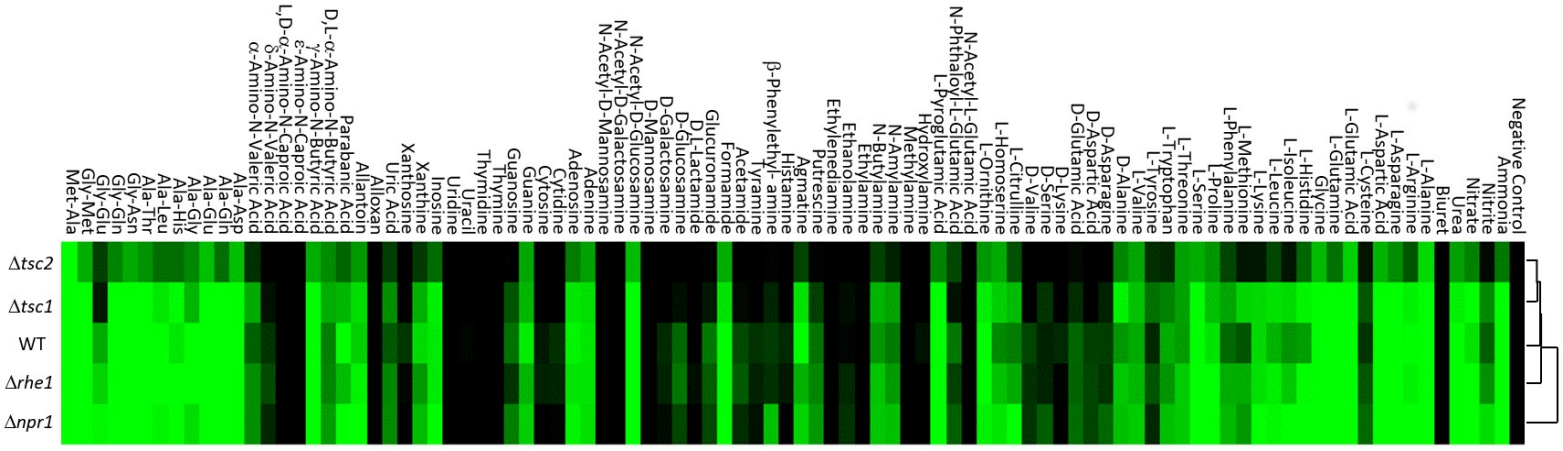
Comparative nitrogen source utilization profiles of *T*. *atroviride* Δ*npr1*, Δ*rhe2*, Δ*tsc1* and Δ*tsc2* mutants and the wild type. Hierarchical clustering of strains cultivated on 95 nitrogen sources and a control using the Biolog Phenotype PM3b nitrogen Microarray system at 25°C for 72 hours. Clustering was done using HCE 3.5 tool with Average Linkage (UPGMA) method implementing Euclidean Distance measure. Heat map represents the mycelial density measured at 600 nm using Tecan Sunrise plate reader with Magellan Tracker software. The color heat map from black to bright green codes for optical density ranging from 0.1 to 0.85.

The nitrogen utilization profiles of the TOR pathway mutants differed from the wild type but also amongst each other. The mycelial growth of the Δ*tsc2* mutant was lower on most of the tested nitrogen sources compared to other strains, including ammonium, L-glutamine, and nitrate (Fig 4). Clustering based on the overall nitrogen utilization patterns of all tested strains after 72 hours of growth revealed a Δ*tsc1* and Δ*tsc2* subcluster suggesting that TSC1 and TSC2 perform similar but specific tasks compared to the other TOR pathway proteins tested (Fig 4). The Δ*npr1-1* and Δ*rhe2* mutants had similar utilization patterns as the wild type. However, Δ*npr1-1* showed enhanced growth in respect to the other tested strains on nitrite, and, together with Δ*rhe2*, on some amino acids such as L-threonine, L-leucine, and L-methionine. Δ*npr1-1* showed reduced growth on ornithine and the dipeptide Ala-Gly and for both, Δ*npr1-1* and Δ*rhe2*, a reduced growth compared to the wild type on xanthine and xanthosine was evident, which, however, was similar to Δ*tsc1* and Δ*tsc2* mutants (Fig 4).

Taken together, these data reveal that the loss of TSC2 most drastically affects nitrogen source utilization and vegetative growth of *T*. *atroviride*. Although deletion of *npr1* or *rhe2* had no or only a minor effect on radial growth on the nitrogen sources tested in plate assays, nitrogen profiling revealed distinct effects of NPR1-1 and RHE2 on the ability of the fungus to assimilate nitrogenous compounds. In addition, clustered nitrogen utilization profiles of Δ*tsc1* and Δ*tsc2* suggest that TSC1 and TSC2 may function as a complex in *T*. *atroviride*, which is in accordance with mammalian and *S*. *pombe* TSC orthologues [24, 42]. In summary, it is evident that the nitrogen source utilization and growth of *T*. *atroviride* is TOR pathway dependent.

### The TOR pathway impacts the mycoparasitic activity of *T*. *atroviride*

**Fig 5:**
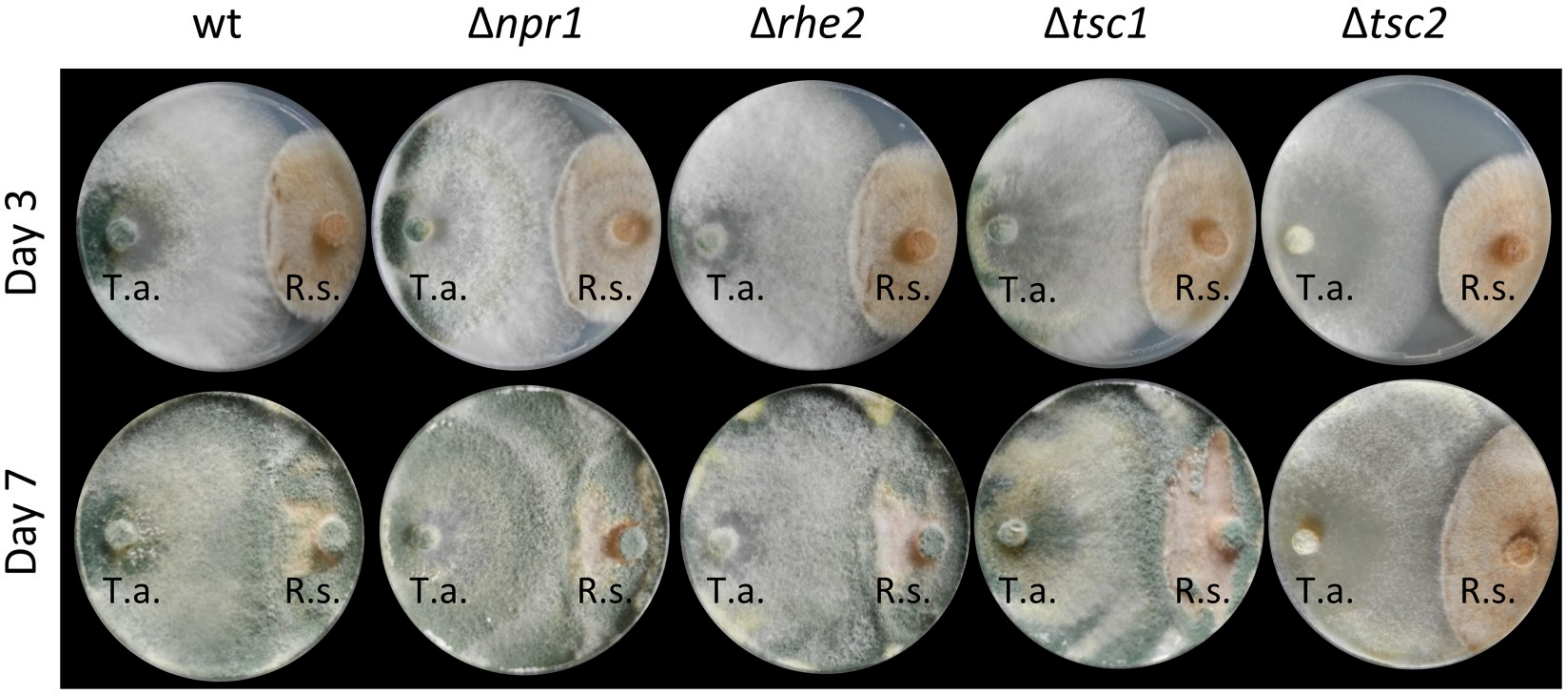
Confrontation assays for assessing the mycoparasitic activity of *T*. *atroviride* Δ*npr1*, Δ*rhe2*, Δ*tsc1* and Δ*tsc2* mutants and the wild type. Fungi were inoculated on opposite sides of a PDA plate (left side: *T*. *atroviride*; right side: *R*. *solani*) and grown at 25°C for 7 days. Pictures were taken after 3 and 7 days.

To assess the role of TOR signaling in *T*. *atroviride* mycoparasitism, the antagonistic behavior of the deletion mutants against *Rhizoctonia solani* (Basidiomycota, Agaricomycetes) as fungal host was monitored in plate confrontation assays on PDA (Fig 5) and on MM with ammonium sulfate (S5 Fig), as these conditions supported growth of both interaction partners. The soil- borne phytopathogen *R*. *solani*, that causes diseases in a wide range of crops, was selected as host due to its agricultural importance. During the early phases of the interaction, in which the mycoparasite grew towards the host and established direct contact, Δ*npr1-1*, Δ*rhe2* and Δ*tsc1* mutants behaved similar as the wild type on both media tested. In contrast, interaction of the Δ*tsc2* strain with the host fungus was delayed. After 7 days, the wild type, Δ*npr1-1* and Δ*rhe2* had almost completely overgrown the *R*. *solani* colony while the Δ*tsc1* mutant showed an only incomplete overgrowth of the host fungus. Loss of *tsc2* most significantly impaired mycoparasitic overgrowth as Δ*tsc2* mutants only unassertively attacked the host or even, upon cultivation on PDA, stopped growing at the interaction border (Fig 5). From these results, we conclude that activated TOR1 resulting from loss of TSC1 or TSC2 negatively affects the mycoparasitic overgrowth activity of *T*. *atroviride*.

### TOR pathway interference affects *T*. *atroviride* secondary metabolite production

*T*. *atroviride* is a prolific producer of secondary metabolites, some of which have antifungal properties and hence are relevant for the mycoparasitic interaction [46]. Since TOR is known to act not only as nutrient sensitive regulator of cell growth but also as a player in nitrogen regulation of fungal secondary metabolism [32], we sought to investigate the effect of TOR signaling on the formation of secreted, nitrogen containing low-molecular weight compounds. To this end, the metabolite profiles of the mutants Δ*rhe2*, Δ*tsc1*, and Δ*tsc2*, that lack TOR pathway regulatory proteins, were compared with those of wild type cultures using an isotope-assisted untargeted metabolomics approach based on liquid chromatography-high resolution mass spectrometry (LC-HRMS/MS). Cultivation of the tested strains on native and uniformly ^13^C-labeled glucose in the presence of native or (^15^NH_4_)_2_SO_4_ as a nitrogen source enabled the assignment of both the global and ^15^N-containing submetabolomes similar to [47, 48]. LC-HRMS measurements and subsequent data evaluation with MetExtract II resulted in a total of 2529 metabolite ions corresponding to 1153 fungal metabolites. Among those, a total of 364 metabolites (represented by 767 features) were found to constitute the N-containing submetabolome (S6 Fig). Detected metabolites were further characterized by their *m/z* values as well as their total number of carbon and nitrogen atoms.

**Fig 6:**
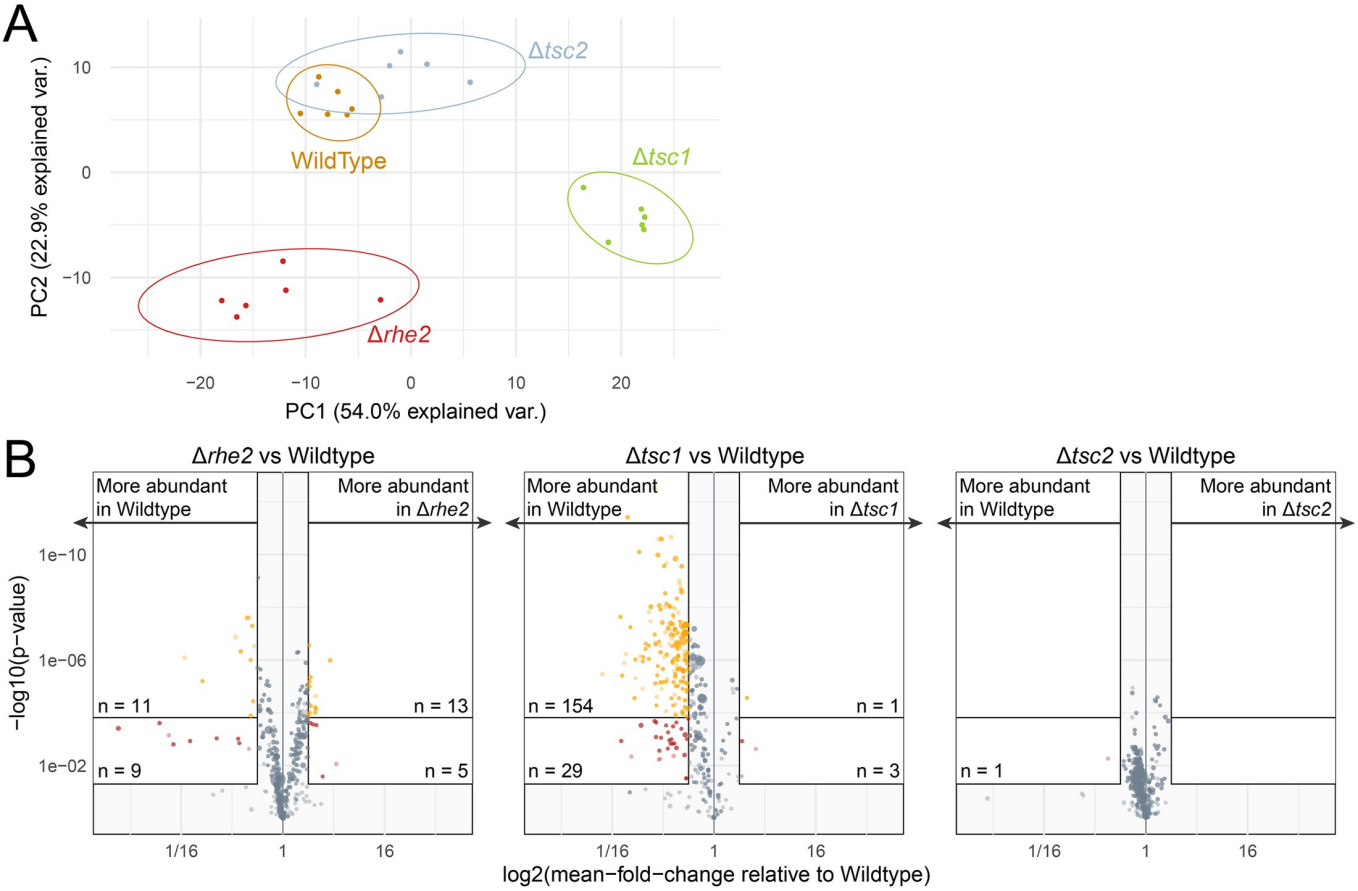
Profiles of low molecular weight substances secreted by *T*. *atroviride* Δ*rhe2*, Δ*tsc1* and Δ*tsc2* mutants and the wild type. Fungal strains were grown in six replicates in minimal medium containing either unlabeled or uniformly labeled (NH_4_)_2_SO_4_ and glucose as nitrogen- and sole carbon source respectively. Culture supernatants were harvested after 14 days of growth at 25°C, analyzed and full scan LC-HRMS chromatograms were processed and filtered for the N-containing submetabolome. (A) Principal component analysis (PCA) scores plot. Clustering of fungal culture samples along PC 1 and PC2 is shown based on the metabolite profiles with 338 N-containing compounds. Ellipses depict the 95% confidence intervals. While samples of the Δ*tsc1* and Δ*rhe2* mutant are clearly separated from each other and the wild type, the Δ*tsc2* mutant samples largely overlap with those of the wild type. (B) Volcano plots illustrating pairwise univariate comparisons of the three mutant strains Δ*rhe2* (left), Δ*tsc1* (middle) and Δ*tsc2* (right) versus the *T*. *atroviride* wild type based on their N-containing submetabolomes. Red dots indicate significantly (p<0.05, FC≥2), those in yellow indicate highly significantly (p<0.00015, FC≥2) differing metabolite abundances, while grey dots indicate not significantly different metabolites.

When the data matrix consisting of 24 samples (6 replicates per strain and 338 N-containing metabolites; outliers removed) was subject to principal component analysis, three distinct clusters of samples confirmed major differences among the metabolite profiles of the tested mutants Δ*rhe2*, Δ*tsc1* and Δ*tsc2*. While Δ*rhe2* and Δ*tsc1* clearly differed from each other and the wild type (Fig 6A), no clear separation between the Δ*tsc2* mutant and the wild type was evident in the PCA score plot pointing to similar metabolite profiles and hence an only minor role of TSC2 in the regulation of *T*. *atroviride* secondary metabolism under the conditions tested. For a more detailed evaluation of the clustering of the investigated fungal samples, metabolite levels were compared pairwise between each of the respective mutants and the wild type (Fig 6B). In cultures of the Δ*rhe2* mutant, a total of 38 metabolites were altered significantly or highly significantly. Of those, 20 metabolites were more abundant in the wild type, while 18 were detected at higher levels in the mutant. In case of Δ*tsc1*, the most pronounced differences were found between the mutant and the wild type. Of the 187 differing compounds, the large majority (n = 183) was produced in significant (n = 29) or highly significant (n = 154) lower amounts by the mutant compared to the wild type. For only four metabolites, higher levels were found in the mutant. In contrast, a comparison of the Δ*tsc2* mutant and the wild type revealed only one single compound to be significantly differing between the two strains. The data suggest a mainly stimulatory effect on the production of secreted N-containing low-molecular weight metabolites exerted by TSC1 in a TSC2-independent manner. RHE2 both positively and negatively governed secondary metabolite production, which altogether suggests the TOR pathway as a key player in the regulation of *T*. *atroviride* secondary metabolism.

### Deletion of *rhe2* renders *T*. *atroviride* hypersensitive to rapamycin and results in reduced RPS6 phosphorylation upon rapamycin treatment

**Fig 7:**
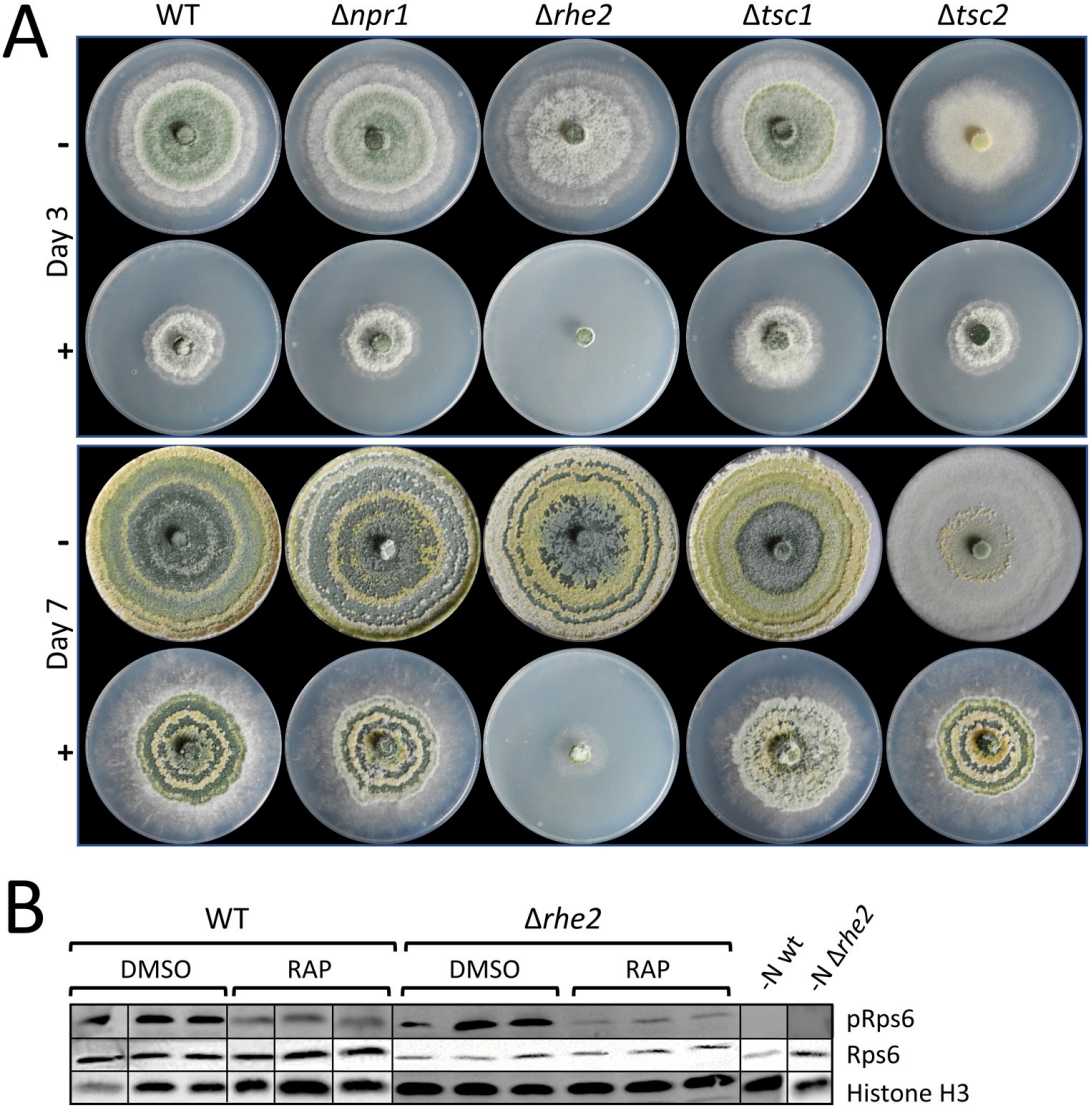
Effect of rapamycin on growth of *T*. *atroviride* Δ*npr1*, Δ*rhe2*, Δ*tsc1* and Δ*tsc2* mutants and phosphorylation of ribosomal protein S6. **(A)** Growth of *T*. *atroviride* wild type and Δ*npr1*, Δ*rhe2*, Δ*tsc1* and Δ*tsc2* mutants on minimal medium with 50 mM ammonium sulfate and 10 μg/ml rapamycin (+). Control samples (-) were amended with equal amount of the solvent DMSO. Plates were incubated at 25°C and pictures were taken after three and seven days. **(B)** Western blot analysis of ribosomal protein S6 (RPS6) phosphorylation in *T*. *atroviride* wild type and the rapamycin hypersensitive Δ*rhe2* mutant after nitrogen starvation for five hours (-N) and subsequent growth in the presence (RAP) or absence (DMSO) of 20 μg/ml rapamycin in minimal media with 50 mM ammonium sulfate for two hours (three biological replicates for both rapamycin treatment and DMSO control are shown). Samples were probed for P-RPS6, total RPS6, and the loading control histone H3 using antibodies anti-phospho-(Ser/Thr) Akt substrate, anti-Rps6, and anti-H3.

We next analyzed the deletion mutants for putative alterations in their rapamycin susceptibility. While growth of Δ*npr1*, Δ*tsc1* and Δ*tsc2* mutants was affected by rapamycin similarly as in the wild type, the Δ*rhe2* mutant showed hypersensitivity as indicated by the inability to form colonies when exposed to the drug (Fig 7A).

To monitor the activity of *T*. *atroviride* TOR1 in response to rapamycin, we analyzed the phosphorylation state of the ribosomal protein S6 (RPS6) by Western blotting as previously described [49, 50]. Consistent with the predicted molecular weight of *T*. *atroviride* RPS6 (Ta_256368; 27.26 kDa), a ~30 kDa band corresponding to phosphorylated RPS6 was visible in samples derived from the wild type when grown in the presence of ammonium sulfate (Fig 7B). Nitrogen starvation led to a complete loss of RPS6 phosphorylation. Rapamycin treatment in the presence of ammonium sulfate resulted in reduced levels of phosphorylated RPS6. The rapamycin hypersensitive Δ*rhe2* mutant reacted to rapamycin with a stronger decrease in RPS6 phosphorylation than the wild type (Fig 7B), while RPS6 largely remained phosphorylated in Δ*tsc1* and Δ*tsc2* mutants upon rapamycin treatment (S7 Fig). These data reveal that, similar to yeast and mammalian cells, analysis of the RPS6 phosphorylation state allows to monitor the activity level of *T*. *atroviride* TOR1. The results further correlate with the high resistance of the *T*. *atroviride* wild type to rapamycin (indicated by the fact that a complete dephosphorylation of RPS6 in response to rapamycin could not be obtained under the conditions tested) and the enhanced rapamycin sensitivity of the Δ*rhe2* mutant.

### The transcriptomic response of *T*. *atroviride* to rapamycin mainly involves metabolism and ribosome/translation associated processes but also mycoparasitism-relevant genes

To gain an overview of the genes and cellular processes regulated by *T*. *atroviride* TOR1, we performed RNA-seq analyses of the wild type as well as the rapamycin hypersensitive Δ*rhe2* mutant after treatment with 20 μg/ml rapamycin or the solvent DMSO as a negative control. More than 260 million filtered reads were obtained of which > 90% could be mapped to the *T*. *atroviride* reference genome. Transcript levels in the rapamycin-treated samples of both strains were compared to their respective DMSO controls and gene expression differences were identified.

**Fig 8:**
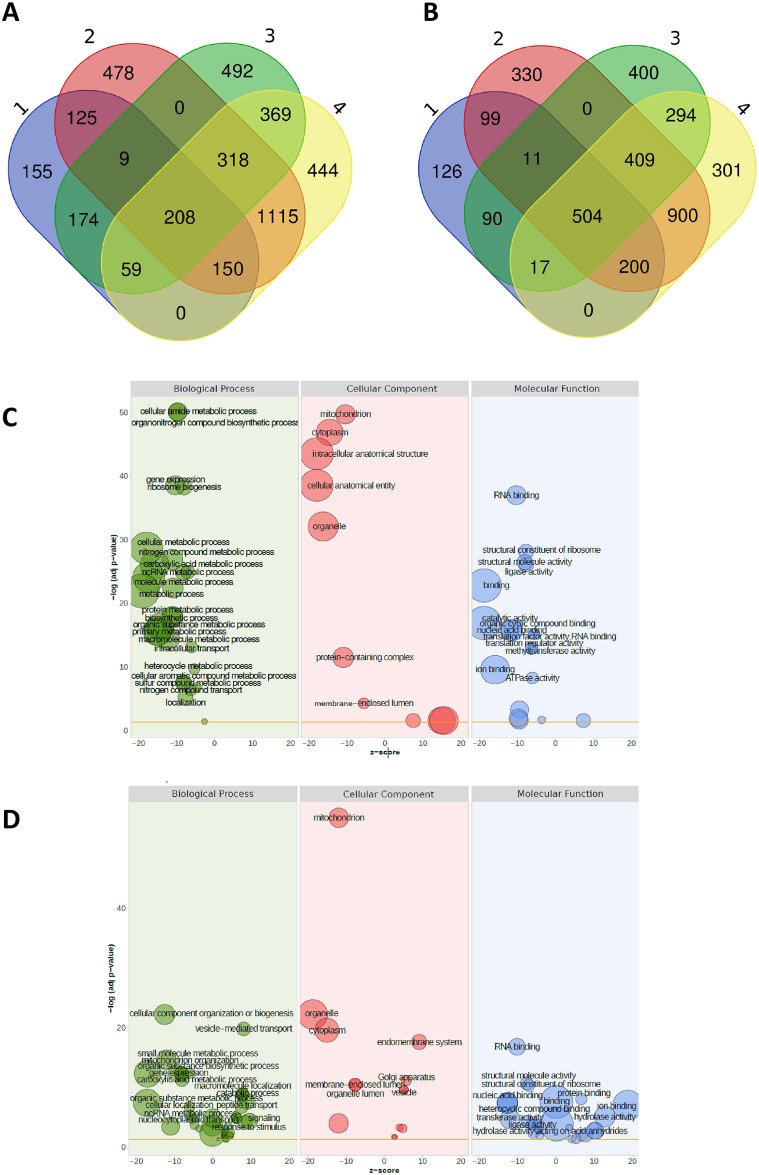
Transcriptional changes due to reduction of TOR1 activity caused by rapamycin treatment as well as by *rhe2* gene deletion. Venn diagrams illustrating the number of differentially expressed genes (DEGs) showing upregulation **(A)** and downregulation **(B)** in the four different comparisons (see S8A Fig) analyzed. The list of DEGs of each comparison is provided in S3 and S4 Files. Bubble plots displaying enriched GO terms of the DEGs emerging from comparisons 1 **(C)** and 3 **(D)**. The x-axis represents the z-score, the y-axis indicates the significance of the term (-log10 adjusted p-value). The area of plotted bubbles is proportional to the number of genes assigned to the GO term.

The gene set affected by rapamycin in the wild type (comparison 1; S8A Fig) comprised 1927 candidates with differential expression (adjusted *p* value threshold of 0.05) of which 1047 were down-regulated and 880 were up-regulated (Fig 8A and 8B). The top ten genes with the strongest rapamycin-triggered down-regulation (based on a list ranked according to log_2_ fold change values) comprised a putative ferric reductase, a terpenoid synthase, a haem peroxidase, a glutamine hydrolysing pyridoxal 5’-phosphate synthase, an amino acid transporter, a putative ATPase, and two G protein-coupled receptors (GPCRs). Differentially expressed genes (DEGs) with the strongest rapamycin-triggered up-regulation coded for a pleiotropic drug resistance protein of the ABC superfamily, a glycoside hydrolase family 89 protein, a methyltransferase, an aromatic amino acid aminotransferase, a carboxypeptidase, a putative monocarboxylate transporter, and an alcohol dehydrogenase superfamily protein (S2 File). 4856 genes emerged as differentially expressed in the Δ*rhe2* mutant upon rapamycin treatment (comparison 2) with 2403 being up- and 2453 down-regulated (Fig 8A and 8B). The top ten DEGs with the strongest rapamycin-triggered down-regulation in the mutant coded for a hydrophobin, an aldehyde dehydrogenase, a mitochondrial ATP synthase, a long-chain-fatty-acid-CoA ligase, a protein required for ribosome biogenesis, and two predicted transporters, while those up-regulated in a rapamycin-triggered manner coded for a predicted GPCR, glycosyltransferase family 8 and 15 proteins, a multicopper oxidase, a small secreted cysteine-rich protein, two putative peptidases, as well as the eliciting plant response-like protein EPL1 (S2 File).

Interestingly, clustering analysis of the DEGs emerging from the four performed comparisons (S8A Fig) revealed that comparisons 1 (WTrap − WT_DMSO_ = wild type response to rapamycin) and 3 (Δ*rhe2*_DMSO_ − WT_DMSO_ = genes differentially expressed between mutant and wild type in the absence of rapamycin) formed a subcluster (S8B Fig). These findings indicate that reduction of TOR1 activity caused by either rapamycin or by *rhe2* gene deletion results in similar transcriptional changes, i.e., 264 shared DEGs (174 commonly up- and 90 commonly down-regulated) (Fig 8A and 8B; S3 and S4 Files).

GO categorization and enrichment analyses showed that the genes involved in the rapamycin response of the wild type are mainly involved in metabolism (including metabolism of nitrogen compounds, small molecules, macromolecules, heterocycles, sulfur compounds), associated to ribosome biogenesis, gene expression and translation, and related to mitochondria-located processes, all of which were down-regulated (Fig 8C). Similar metabolic, ribosome- and mitochondria-associated processes were down-regulated in comparison 3, but, in addition, genes enriched in up-regulation of e.g. vesicle-mediated transport, signaling, cellular response to stimulus, and macromolecule modification resulted from *rhe2* gene deletion (Fig 8D). Among the top ten genes being down-regulated in the Δ*rhe2* mutant compared to the wild type under normal growth (DMSO solvent control, i.e. absence of rapamycin; comparison 3) were, among *rhe2* itself, candidates encoding a glycoside hydrolase family 16 protein, two WSC domain-containing proteins, three secreted cysteine-rich proteins, a putative GPCR, and a putative polyketide synthase (S2 File). Top ten DEGs being up-regulated in the Δ*rhe2* mutant compared to the wild type included genes coding for a pleiotropic drug resistance protein of the ABC superfamily, an aquaporin, a polyketide synthase domain-containing protein, a glycoside hydrolase family 89 protein, and an AAA+-type ATPase (S2 File).

Taken together, transcriptome analysis showed that, similar to other eukaryotes, *T*. *atroviride* TOR1 controls the expression of genes coding for ribosomal components and is a central hub of cellular metabolism. In addition, several DEGs with known or supposed roles in mycoparasitism and secondary metabolism were among the identified TOR1 targets. The transcriptional changes in the wild type in response to rapamycin furthermore turned out to be similar to the transcriptional changes caused by the deletion of *rhe2* evidencing a role of RHE2 in TOR1 activation.

## Discussion

The TOR kinase pathway is the main nutrient-responsive signaling route in eukaryotes and regulates essential cellular processes such as growth, metabolism and ribosome biogenesis. TOR signaling is well studied in yeasts such as *S*. *cerevisiae*, *S*. *pombe* and *C*. *albicans* as well as in mammalian cells. In these systems, TOR is active and promotes the synthesis of new ribosomes under nutrient (especially nitrogen and amino acid) sufficiency, while TOR activity declines, thereby leading to a reduction in ribosome biogenesis and growth, when nutrients or energy are limited or upon TOR kinase inhibition by respective drugs (reviewed in [51, 52]. Despite the importance of mycoparasitic *Trichoderma* species as biocontrol agents in plant protection and decades of research on the mechanisms of mycoparasitism, data on how nutrient-sensing pathways affect the lifestyle and mycoparasitic activity of these fungi are scarce.

To our knowledge, we here demonstrate for the first time that the mycoparasite *T*. *atroviride* possesses a functional TOR signaling pathway that governs processes not only required for vegetative growth and metabolism, but also for mycoparasitism. In addition to the conserved TOR kinase itself, we identified additional components of the TOR complexes 1 (TORC1) and 2 (TORC2) and functionally characterized putative regulatory proteins of the TOR signaling pathway. *S*. *cerevisiae* and *S*. *pombe* are among the best studied fungal organisms regarding TOR signaling; however, they differ to most other species, including *T*. *atroviride*, by encoding two distinct TOR kinase paralogs [38]. In addition to a single TOR protein (TOR1), *T*. *atroviride* possesses orthologues of the upstream TOR kinase regulators TSC1 and TSC2 as well as the Rheb GTPase which in *S*. *pombe* and mammalian cells regulate the activity of TORC1 [53, 54]. Based on *S*. *cerevisiae* Npr1 and its three *F*. *fujikuroi* orthologues, three NPR1 candidates could be identified in the *T*. *atroviride* genome. *T*. *atroviride* NPR1-1 emerged as the protein with the highest similarity to yeast Npr1 and was able to partially complement the growth defect of a *S*. *cerevisiae npr1Δ* mutant on ammonium and urea (Fig 2), a function being comparable to *F*. *fujikuroi* FfNpr1-1 [43]. The Npr1 serine/threonine protein kinase is controlled by TORC1 and regulates the activity of permeases for nitrogenous substances in *S*. *cerevisiae*, hence is necessary for nitrogen uptake in yeast cells grown on non-preferred nitrogen sources [4–7, 55, 56]. Deletion of *npr1-1* in *T*. *atroviride* resulted in only subtle phenotypic changes, such as a slightly reduced growth on agar plates in the presence of nitrate or L-proline as sole nitrogen sources (Fig 3). Similar results have previously been reported for *F*. *fujikuroi*, where *FfNPR1-1*, *FfNPR1-2* and *FfNPR1-3* deletion mutants did not show significant growth defects on the tested nitrogen sources tempting the authors to summarize that the Npr1-like kinases have no major impact on the activity of the amino acid permease Gap1 in *F*. *fujikuroi* [43].

*T*. *atroviride* showed an unexpected resistance to rapamycin, an allosteric inhibitor of mainly TORC1. Rapamycin did not entirely inhibit *T*. *atroviride* growth even at concentrations of up to 60 μg/ml (Fig 1). A similar low rapamycin sensitivity has been reported for the zygomycete *Mucor circinelloides* [57] and recently also for the industrial cellulase producer *Trichoderma reesei* Rut-C30 [58]; in *S*. *pombe*, TORC1 is as well only incompletely inhibited by rapamycin [59]. In contrast, species of the order Hypocreales such as *Fusarium graminearum*, *F*. *fujikuroi*, *F*. *oxysporum* and *Verticillium dahliae* exhibited a significantly higher rapamycin sensitivity with concentrations in the range of 5 ng– 200 ng rapamycin per ml PDA being effective in inhibiting fungal growth [31–33, 60]. Interestingly, *T*. *atroviride* was more sensitive to the ATP-competitive inhibitor Torin1 to which it responded even stronger than *V*. *dahliae* [60], although a complete shutdown of fungal growth could not be obtained under the tested conditions. The combined use of rapamycin and torin1, the latter being active also against TORC2 and able to inhibit the rapamycin-resistant TORC1 activity [20, 21], resulted in a nearly complete inhibition of *T*. *atroviride* growth though. This suggests that both TOR kinase inhibitors are needed to reduce *T*. *atroviride* TOR1 activity to a level below that needed to support growth, probably because of TORC1 being only partially sensitive to rapamycin. Interestingly, a gene encoding a pleiotropic drug resistance protein of the ABC superfamily was among the candidates with the strongest rapamycin-triggered up-regulation in the *T*. *atroviride* wild type suggesting that the high resistance of the fungus to rapamycin could be due to its efficient detoxification by transmembrane export. In fact, various *Trichoderma* strains are able to resist and degrade synthetic chemicals and toxins and *T*. *atroviride* strain P1 even represents a fungicide resistant isolate that is compatible with certain fungicides used in agriculture [61, 62].

In mammalian and yeast cells, TOR signaling controls the phosphorylation of ribosomal protein S6 (Rps6) and thereby activates protein synthesis [17, 63, 64]. A collaboration in regulating Rps6 phosphorylation between TOR and protein kinase A (PKA), which mainly responds to carbon sources, has been shown for *Candida albicans* [50]. To monitor the activity of the TOR pathway in *T*. *atroviride* and to prove the inhibitory action of rapamycin on TOR1, we used RPS6 phosphorylation as a biochemical read-out. Starvation for nitrogen led to a complete loss of RPS6 phosphorylation, while rapamycin treatment resulted in reduced levels of phosphorylated RPS6 (Fig 7). While these data are correlated to the partial sensitivity of *T*. *atroviride* TOR1 to rapamycin, at the same time they imply a rapamycin-resistant residual activity. Rps6 phosphorylation was only marginally reduced by treatment with rapamycin also in *S*. *pombe* [21] and TORC1 and TORC2 have been shown to work in synergy in the regulation of Rps6 phosphorylation in *S*. *cerevisiae* [65].

The Rheb GTPase is conserved from yeast to human and plays important roles in TOR activation. Our results show that deletion of the single Rheb orthologue-encoding gene (*rhe2*) in *T*. *atroviride* results in an enhanced sensitivity to rapamycin, which was not only evident in growth assays but also on the level of RPS6 phosphorylation (Fig 7), suggesting that RHE2 is associated to the TOR signaling pathway also in *T*. *atroviride*. Accordingly, *RHB1* gene deletion in *C*. *albicans* led to enhanced rapamycin sensitivity [66]. The observed normal growth in the presence of rich nitrogen sources or in complete media (PDB and PDA), but the slightly reduced growth on agar plates with the poor nitrogen sources nitrate and proline resulting from loss of *rhe2* in *T*. *atroviride*, is consistent with the phenotype previously described for the *A*. *fumigatus* Δ*rhbA* mutant [28]. Interestingly, *rhbA* deletion resulted in reduced virulence of *A*. *fumigatus* in a mouse model of invasive aspergillosis [28], while the *T*. *atroviride* Δ*rhe2* mutant was as effective as the wild type in attacking the plant pathogen *R*. *solani* (Fig 5). Considered in context with the observed impaired antagonism of the *T*. *atroviride* Δ*tsc1* and Δ*tsc2* mutants (the TSC complex is supposed to negatively regulate RHE2-TOR signaling) a picture emerged that evidences a negative regulatory role of TOR1 activity in *T*. *atroviride* mycoparasitism. Although in the case of Δ*tsc1*, the effect on mycoparasitism could be indirect due to the reduced growth of this mutant under the conditions tested, this model is in accordance with previous studies revealing that *T*. *atroviride* faces stress from nitrogen limitation and up-regulates genes for non-preferred nitrogen source assimilation when confronted with a living host fungus [34, 35]. Further support comes from reports on *S*. *pombe* where loss of Rhb1 function and disruption of TORC1 mimic a nitrogen starvation phenotype including activation of nitrogen-starvation gene expression [67–69].

In mammalian and *S*. *pombe* cells, TSC1 and TSC2 have been shown to form the heterodimeric TSC complex that negatively regulates the activity of Rheb and TORC1 by catalyzing GTP hydrolysis on Rheb [24, 42]. This activity is derived from the GTPase-activating domain of TSC2, while TSC1 is supposed to enhance and stabilize TSC2 activity but also bind other proteins in addition to TSC2 [70–72]. In mammals, the TSC complex acts as tumor suppressor that inhibits mTORC1 to limit undesirable cell growth [73] but also physically associates and positively regulates mTORC2 in a Rheb-independent manner [42]. Accordingly, disruption of the TSC complex leads to hyperactivated TORC1 signaling in *S*. *pombe* that abandons the scavenging of nitrogen sources [24, 25]. Deletion of either *tsc1* or *tsc2* in *T*. *atroviride* resulted in reduced radial growth on agar plates, which was especially evident on MM with nitrate or proline as nitrogen sources and more pronounced in Δ*tsc2* than Δ*tsc1* mutants (Fig 3). Although loss of TSC2 most drastically affected growth and nitrogen source utilization among all the mutants included in this study, the nitrogen utilization profiles of Δ*tsc1* and Δ*tsc2* mutants derived from Biolog analysis clustered together. These data indicate similar roles of the TSC1 and TSC2 proteins in regulating *T*. *atroviride* nitrogen source-dependent growth, which is in accordance with their function as components of the TSC complex. Nonetheless, Δ*tsc1* and Δ*tsc2* mutants also differed, which was particularly evident in their secondary metabolite profiles. While deletion of *tsc2* only marginally affected the production of secreted N-containing low molecular weight substances, evidence for a mainly stimulatory, but TSC2-independent role in secondary metabolite production emerged for TSC1 in *T*. *atroviride*. TSC2-independent functions of TSC1 as well as noncanonical, i.e. Rheb- and TOR-independent, functions of the TSC genes have been reported in multiple organisms [72].

Despite its high resistance to rapamycin, *T*. *atroviride* experienced a significant transcriptional reprogramming in response to this drug. TOR1 inhibition by rapamycin resulted in the downregulation of various metabolic processes and processes associated to ribosome biogenesis, gene expression and translation (Fig 8). The negative effect of a reduced TOR1 activity on these cellular processes was also evident in the transcriptome of the Δ*rhe2* mutant, supporting the role of the RHE2 GTPase as an activator of TOR1. Genes with the strongest differential regulation due to TOR1 inhibition comprised proteins involved in ribosome biogenesis and energy production, several transporters of the MFS superfamily and amino acid transporters. These findings are in accordance with studies in mammalian and yeast cells where the transcript levels of ribosomal components are repressed by rapamycin and where TOR has been implicated in the transcriptional regulation of amino acid permeases [74, 75], thereby supporting a highly conserved role of the TOR kinase signaling pathway also in *T*. *atroviride*. Gene ID246378, encoding a candidate pleiotropic drug resistance protein of the ABC superfamily, showed the highest rapamycin-trigged upregulation in the *T*. *atroviride* wild type (log2FC 14.6) in our study. This ABC transporter could be responsible for transporting rapamycin out of the cells and thereby cause the high rapamycin resistance of *T*. *atroviride*. According to the rapamycin hypersensitivity of the Δ*rhe2* mutant, ID246378 was upregulated by rapamycin to a considerably lesser extent (log2FC 4.1) in the mutant than in the wild type. The TOR target genes with the highest differential regulation also included candidates with a putative function in mycoparasitism. These were, for example, terpenoid and polyketide synthases involved in secondary metabolite production, virulence associated PTH11-type GPCRs, that are necessary for appressorium formation in the plant pathogen *Magnaporthe oryzae* [76], a putative ferric reductase that in *Crypotococcus neoformans* is involved in iron acquisition and virulence [77], several peptidases such as serine carboxypeptidases and metallopeptidases [78], glycoside hydrolases of families GH15, GH 16, and GH 89 including N-acetylglucosaminidase [79], as well as small secreted cysteine-rich proteins including eliciting plant response protein EPL1 that acts as an elicitor for the induction of plant defense responses against pathogens [80, 81]. The expression of genes for glycoside hydrolases involved in plant cell wall degradation is affected by rapamycin in the plant pathogen *V*. *dahliae* [60] and the cellulase producer *T*. *reesei* [58], which is similar to our findings and evidences a role of TOR1 in the regulation of carbohydrate active enzyme expression.

To our knowledge, this is the first comprehensive study of TOR kinase signaling in a mycoparasitic fungus. Biochemical inhibition of TOR1 as well as a reverse genetics approach for elucidating the functions of TOR1 regulatory proteins revealed that the TOR signaling pathway plays a critical role in regulating growth by targeting genes being similar to those in yeast model organisms and mammals. In addition, several mycoparasitism-relevant genes and processes turned out to be under control of TOR1, which enhances our knowledge on the regulation of the mycoparasitic activity of *T*. *atroviride*. A detailed understanding of the signaling pathways that govern the responses of *T*. *atroviride* to environmental cues, including those derived from host fungi, is of utmost importance for the enhancement and targeted application of this biocontrol agent.

## Materials and methods

### Strains and culture conditions

*T*. *atroviride* strain P1 (ATCC 74058) was used throughout this study as the wild type and all mutants were derived from this strain. Fungal strains were cultivated at 25°C using a 12 hours light/dark cycle on either potato dextrose agar (PDA; Merck, Germany) or minimal medium (MM) [82]. For dual confrontation assays, *T*. *atroviride* was co-cultivated with the agriculturally important plant pathogen *Rhizoctonia solani* (pathogenic isolate obtained from the collection of the Department of Agricultural Sciences, Università degli Studi di Napoli “Federico II,” Naples, Italy) as host fungus on PDA or MM with 50 mM ammonium sulfate as sole nitrogen source. Media were supplemented with rapamycin (Selleckchem, Houston, USA; 100 mg/ml stock solution in DMSO) or Torin1 (Selleckchem; 4 mg/ml stock solution in DMSO) where indicated. For assessing radial growth rates, colony growth on agar plates was recorded twice a day for up to 10 days. For the analysis of biomass formation, 250 ml Erlenmeyer flasks containing either 50 ml of potato dextrose broth (PDB; Merck, Germany) or MM with either 10 mM ammonium sulfate, glutamine, N-acetyl-glucosamine, or proline as sole nitrogen source were inoculated with 10^6^ fungal spores/ml medium (final concentration). After 72 hours of incubation at 25°C and 200 rpm, fungal biomass was determined by recording mycelial dry weight. *Escherichia coli* strains JM109 (Promega, Madison, Wisconsin) and Stellar^®^Top10 (Clontech, Takara Bio Europe, Saint-Germain en Laye, France) were used for plasmid construction and amplification.

### Generation and verification of *T*. *atroviride* gene deletion mutants

*T*. *atroviride* gene deletion mutants were generated by transforming fungal protoplasts with linearized gene deletion cassettes containing the *E*. *coli hph* gene under control of the *T*. *reesei gpd1* promoter and *A*. *nidulans trpC* terminator as selection marker. Deletion vectors were constructed using yeast homologous recombination as previously described [83]. To this end, gene specific primers (S5 File) were designed for PCR amplification of ~1-kb of the up- and downstream flanking non-coding regions of the respective gene of interest with overhangs to the *hph* resistance cassette using *T*. *atroviride* genomic DNA as template. Transformants were selected on PDA containing 200 μg/ml hygromycin B (Calbiochem, California, USA) and purified to mitotic stability by three rounds of single spore isolation. Homologous integration of the deletion constructs at the target locus and gene deletion was confirmed by PCR using one primer binding in the genomic region flanking the integration site and a second primer binding inside the *hph* gene (S5 File). The copy number of the deletion cassettes in the transformants was analyzed by Southern and qPCR and strains comprising one copy of the deletion cassette at the target locus were used for further experiments.

Southern blotting was performed on total genomic DNA (isolated with E.Z.N.A.^®^ Tissue DNA Kit, Omega Bio-tek, Norcross, GA 30071, USA) digested with the restriction enzymes *Sac*I or *Nhe*I (Thermo Scientific, Waltham, Massachusetts, USA) using DIG-labelled probes for the *hph* gene and following the manufacturer’s instruction (Roche, Basel, Switzerland). *Hph* copy number analysis of Δ*rhe2*, Δ*npr1*, Δ*tsc1* and Δ*tsc2* mutants was performed by quantitative PCR as described [84] using total genomic DNA and primers HPH_F1 and HPH_R1 (S5 File). *Sar1* was used as reference gene [85] and strain Δ*tsc1* 11blC with one copy of the *hph* marker cassette was used for normalization. The PCR protocol consisted of an initial denaturation step (3 min at 95°C) followed by 40 cycles of denaturation (15 sec at 95°C), annealing (30 sec at 65°C) and extension (72°C for 40 sec) followed by a melting curve analysis. All samples were analyzed in three independent experiments with three replicates in each run.

### Biolog phenotype array analysis

The Biolog PM3b MicroPlate assay (Biolog Inc., Hayward, CA) which comprises 95 wells with different nitrogenous compounds and a well with water as negative control was used to investigate growth rates and comparative nitrogen source utilization of the *T*. *atroviride* wild type and the TOR pathway mutants. Commercially available FF inoculation fluid (Biolog #72106) containing 16 ml of sterile ’gelling’ inoculating fluid (0.25% Phytagel and 0.03% Tween 40) was supplemented with 50 mM glucose. Conidia were collected from the tested *Trichoderma* strains as previously described [86] and inoculated to the test tubes to the final concentration of 2x10^6^ spores/ml. The microplates were incubated in darkness at 25°C. Optical density at 600 nm was measured every 24 hours for up to 96 hours using a Tecan Sunrise microplate reader, which measures the turbidity and reflects biomass production on the tested substrate. For comparative analyses, OD_600_ values from the “72 hours” time point were used and quantitatively illustrated using the Hierarchical Clustering Explorer 3 (HCE3) [87] by applying the hierarchical clustering algorithm with average linkage and Euclidean distance measure. All measurements were performed in triplicates.

### Western blotting

For Western blot analysis, fungi were cultivated in at least three biological replicates as described above. Harvested mycelia were ground in liquid nitrogen and homogenized in extraction buffer (20 mM Tris-HCl, pH 8; 110 mM KCl; 10% (v/v) glycerol; 5mM EDTA; 1 μM pepstatin A; 1 mM Pefablock SC (Sigma-Aldrich, St. Louis, USA)). After centrifugation (16,000 g, 30 min), the liquid phase was collected and the protein concentration determined according to Bradford (ROTI^®^Quant; Roth, Karlsruhe, Germany). ~50 μg of protein were separated by SDS-PAGE and blotted onto nitrocellulose blotting membranes (Amersham Protran, 0.2 μm, 80 x 90 mm, GE Healthcare, Chicago, Illinois, USA). After blocking with 4% BSA, membranes were incubated with the respective antibodies phospho-(Ser/Thr) Akt substrate (#9611, Cell Signaling Technology, Danvers, Massachusetts, USA), Rps6 (#ab70227, abcam, Cambridge, UK), and histone H3 used as loading control (#ab1791, abcam) followed by application of the secondary antibody Goat Anti-Rabbit IgG H&L (#ab175773, abcam). The blots were imaged using a G:box station (Syngene, Cambridge, UK). Conserved TOR-derived phosphorylation sites are present in *T*. *atroviride* RPS6 (Ta_256368; https://mycocosm.jgi.doe.gov/Triat2/Triat2.home.html; predicted molecular weight 27.26 kDa) at Ser235 and Ser236.

### Metabolite profiling

*T*. *atroviride* wild type as well as Δ*tsc1*, Δ*tsc2* and Δ*rhe2* mutants were grown on PDA plates until the cultures conidiated. From each strain, 2x10^3^ spores/ml were inoculated in non-treated, non-coated 24-well plates (Eppendorf Cell Culture Plate) filled with 2 ml/well liquid SM minimal medium [79]. D-glucose (10 g/L) was used as sole carbon source and (NH_4_)_2_SO_4_ (10 mM) as sole nitrogen source. Three media were tested in parallel: unlabeled medium with native D-glucose and (^14^NH_4_)_2_SO_4_, ^13^C-labeled medium with U-^13^C glucose (Sigma Aldrich, St. Louis, Missouri, USA), and (^14^NH_4_)_2_SO_4_, and ^15^N-labeled medium containing native glucose and (^15^NH_4_)_2_SO_4_ (Sigma Aldrich). Each cultivation variant was prepared in six replicates. Incubation was carried out at 25°C in static semi-dark conditions. To estimate fungal growth, OD_750_ of the standing cultures was measured before harvesting, by using an EnSpire^®^ Multimode Plate Reader (PerkinElmer, Waltham, Massachusetts, USA). The culture supernatants were collected after 14 days, quenched with 66.6% (v/v) acetonitrile (ACN, Roth, Graz, Austria) and centrifuged at 20,000 × g for 10 min at 4°C. For both ^13^C-labeled and ^15^N-labeled supernatants, two types of pooled internal standards were prepared: to maximize metabolome coverage, the six labeled culture replicates were pooled for every strain separately and quenched by addition of ACN (1:2 (v/v) supernatant:ACN). For statistical analysis, a global pool across all strains was prepared. For metabolite detection, native supernatants were mixed 1:3 (v/v) with the respective ^13^C- or ^15^N-labeled pools resulting in a final ratio supernatant:ACN of 1:1 (v/v) while the supernatant consisted of a 1:1 (v/v) mix of native and labeled supernatants. This was carried out for every strain separately. For subsequent statistical data evaluation, a 200 μl-aliquot of each native replicate (^12^C-/ ^14^N conditions) was added to 600 μl of ^13^C-labeled pool. For QC purposes, 50 μl of each measurement solution were combined to give an aggregate sample used to monitor LC-HRMS and data processing performance. Negative controls were prepared for each strain by dilution of the respective ^13^C- or ^15^N-labeled pools or the native pool with the appropriate amount of H_2_O or acetonitrile. All samples were then analyzed with HPLC-high-resolution mass spectrometry (HRMS) in the full scan mode with the method described by [88].

For data processing, the measured LC-HRMS raw data files were converted into the mzXML format using msConvert from Proteowizard ([89]; version 3.0.19166-cc86d1f56). The files were then processed with the AllExtract module of MetExtract II [90]. In brief, the software searched for pairs of chromatographic peaks, originating from co-eluting native and ^13^C- or ^15^N-labeled metabolite ions. Both forms had to show distinct mirror-symmetric isotopolog patterns and perfectly coelute with highly similar chromatographic peak shapes. Filtered metabolite ions were aligned across all samples obtained for either the ^13^C- or ^15^N-labeled cultures. Parameter settings of MetExtract II were: Intensity threshold for M and M’: 10000 counts; Start RT: 3 minutes, End RT: 36 minutes; Wavelet min/max scale: 3; EIC extraction: ±5 ppm; Maximum intensity abundance error: ± 0.15; Minimum peak correlation: 0.85 (Pearson correlation); Maximum clustering error: max. ±8 ppm; Number of isotopologs checked: 2; Settings for the integration of the ^13^C- and the ^15^N-labeled culture-derived features: Maximum allowed *m/z* deviation: 5 ppm; Maximum tolerated retention time shift: ± 0.10 minutes.

All statistical analyses were implemented in the R programming language for statistical computing ([91]; version 3.5.3). Only the most abundant features (i.e., ion) per metabolite were used for statistical analysis, which accounted to 364 N-containing metabolites. Of these, 26 metabolites were removed as these were not detected in at least three culture samples. Only the abundances of the native, monoisotopic features were utilized. Missing values were replaced by an abundance of 10,000. The imputed data matrix was auto-scaled. For univariate comparison, a global significance threshold of α = 0.05 and fold change (FC) ≥ 2.0 were used, while Sidak multiple testing correction (α = 0.00015) and FC ≥ 2.0 were used to classify metabolites as highly significantly different between the tested experimental conditions. For the heatmap illustration, the squared Euclidian distance and the Ward linkage method were applied.

### Yeast complementation assay

*T*. *atroviride npr1* was amplified from cDNA as template using primers TaNPR1_cDNA_fw and TaNPR1_cDNA_rev (S5 File). Primers YNpr1_Pro_fw and YNpr1_Pro_rev were used to amplify the promoter and primers TaNPR1_Ter_fw and TaNPR1_Ter_rev (S5 File) to amplify the terminator regions of the *S*. *cerevisiae npr1* gene using genomic DNA of strain 23344c as template. Fragments were combined in vector YEplac195 [92] using the NEB^®^ PCR Cloning Kit (New England Biolabs, Ipswich, UK) and the final plasmid (YEplac195-TaNpr1) was verified by sequencing. Yeast cells, the strains 23344c (*ura3*) and PVV357 (*npr1*Δ *ura3*) [8], were transformed with the indicated plasmids, as described previously [93]. For growth tests, cells were grown in minimal buffered (pH 6.1) medium containing 3% glucose as the carbon source and glutamate 0.1%, ammonium 1 mM, proline 0.1%, urea 0.1% or 1 mM, citrulline 0.05% or tryptophan 0.05% as the nitrogen sources [94].

### Transcriptome analysis by RNA sequencing

*T*. *atroviride* was pre-cultivated for 48 hours in MM with 50 mM ammonium sulfate on a rotary shaker at 25°C starting from an inoculum of 2x10^6^ spores/ml. Washed fungal biomass was then transferred into MM without nitrogen and starved for 5 hours before replacement to fresh MM with 50 mM ammonium sulfate. Three replicates each were treated with 20 μg/ml rapamycin or an equal volume of DMSO for 2 hours. Total RNA was isolated using RNeasy Plant mini kit (Qiagen, Venlo, Netherlands), following manufacturer’s instructions. Final elution was performed in Tris 10 mM, pH 7 (Thermo Fisher Scientific, Waltham, Massachusetts, USA). Illumina TruSeq mRNA library preparation, sequencing and differential gene expression analyses were performed by Microsynth (Balgach, Switzerland) according to their dedicated analysis pipeline. Obtained sequence reads were mapped to the *T*. *atroviride* genome (https://mycocosm.jgi.doe.gov/Triat2/Triat2.home.html) using software Bowtie 2 [95] and Differentially Expressed Genes (DEGs) were identified using DESeq2 [96]. Read counts were normalized, the variance calculated based on the three biological replicates per condition, and statistical testing applied for the identification of DEGs that are significantly up- or downregulated. Four sets of DEGs were identified: 1. comparison of *T*. *atroviride* wild type (WT) transcriptomes when grown in presence of DMSO (solvent control) *versus* growth in presence of rapamycin; 2. comparison of transcriptomes of the Δ*rhe2* mutant grown in presence of DMSO (solvent control) *versus* growth in presence of rapamycin; 3. comparison of transcriptomes of the WT grown in presence of DMSO *versus Δrhe2* grown in presence of DMSO; 4. comparison of transcriptomes of the WT grown in presence of rapamycin *versus* Δ*rhe2* grown in presence of rapamycin (S6 Fig). Identified DEGs were considered as significant if *p*-adjusted value was ≤ 0.05 and were visualized as heatmap generated by using R package “*ggplot2”*. In order to identify shared and common DEGs among comparisons, Venn diagrams showing intersections of the four comparisons were generated through the R package “*ggvenn”*, and shared/common set of DEGs were listed. DEGs were associated by their IDs in the reference genome and the sequences were annotated with Blast2GO v.5 [97] to determine the related Gene Ontology (GO) terms. At the same time, all gene models of *T*. *atroviride* reference genome were annotated through Blast2GO to obtain an updated functional annotation. Fisher enrichment tests for significant DEGs obtained from the four comparisons were performed with Blast2GO to search for significant differences (False Discovery Rate ≤ 0.05) in frequencies of GO terms compared to all *T*. *atroviride* gene models (over representation). Results of Fisher enrichment test was slimmed in REVIGO [98]. The R package "*GOplot*” v. 1.0.2 was used to visualize the enriched GO terms in the four comparisons as bubble plots. For Fisher enrichment results, the z-score for each enriched category was calculated with the following formula: *zscore* = (*up*−*down*)/√*count*, where ‘*up*’ and ‘*down*’ are the number of assigned genes up-regulated (logFC > 0) or down- regulated (logFC < 0), respectively, and ‘*count*’ is the number of genes assigned to a term.

### Identification of TOR pathway components

*T*. *atroviride* orthologues of proteins associated with the TOR signaling pathway were identified by BLASTp searches of the *T*. *atroviride* genome database (https://mycocosm.jgi.doe.gov/Triat2/Triat2.home.html) using default settings and the following queries: *S*. *cerevisiae* Tor1p (*Saccharomyces* Genome Database, SGD: YJR066W), *S*. *cerevisiae* Tor2p (SGD: YKL203C), *S*. *cerevisiae* Kog1p (SGD: YHR186C), *S*. *cerevisiae* Lst8p (YNL006W), *S*. *cerevisiae* Avo1p (SGD: YOL078W), *S*. *cerevisiae* Avo2p (SGD: YMR068W), *S*. *cerevisiae* Avo3p (SGD: YER093C), *S*. *cerevisiae* Rhb1p (SGD: YCR027C), *S*. *cerevisiae* Npr1p (SGD: YNL183C), *F*. *fujikuroi* Ff Npr1-1, FfNpr1-2 and FfNpr1-3 [43], *S*. *pombe* Tsc1 (PomBase: SPAC22F3.13) and Tsc2 (PomBase: SPAC630.13c).

## Supporting information

S1 File **(S1 Table) Mean ± standard deviation (cm) of colony diameter of *T*. *atroviride* wild type on PDA or minimal medium with 50 mM ammonium sulfate amended with rapamycin, torin1 or a rapamycin and torin1 combination as shown in** Fig 1. Values which do not share at least one letter are statistically different within each column according to Tukey’s test (ANOVA). **(S2 Table)** Putative TOR pathway components encoded in the *T*. *atroviride* genome. Protein IDs were assigned by reciprocal best hit Blast using the given queries retrieved from the *S*. *cerevisiae* genome database (SGD; http://www.yeastgenome.org), the *S*. *pombe* genome database (PomBase; https://www.pombase.org), or [43] in case of *F*. *fujikuroi*. **(S3 Table)** Biomass (mg) of *T*. *atroviride* wild type and Δ*npr1*, Δ*rhe2*, Δ*tsc1* and Δ*tsc2* mutants grown for 72 h in shake cultures in PBD or minimal medium amended with either 10 mM ammonium sulfate, L-glutamine or L-proline. Values which do not share at least one letter are statistically different within each row according to Tukey’s test (ANOVA).
(XLSX)

S2 File List of top 10 differentially expressed genes in the four comparisons analyzed. (XLSX)

S3 File List of up-regulated genes in the different comparisons shown in the Venn diagram (Fig 8A). (XLS)

S4 File List of down-regulated genes in the different comparisons shown in the Venn diagram (Fig 8B). (XLS)

S5 File Primers used for generation of transformation cassettes, genotypic verification, and *npr1* complementation. (XLSX)

S1 Fig **(A)** Southern blot analysis to confirm single copy integration of the *hph* deletion cassette in the Δ*tsc1- 11bIC* mutant. Genomic DNA was digested with *Sac*I or *Nhe*I followed by hybridization with a DIG-labelled probe of the *hph* gene. **(B)** qPCR analysis of *hph* copy number in different Δ*rhe2*, Δ*npr1*, Δ*tsc1* and Δ*tsc2* mutants and the wild type control. Fold changes were normalized to the single copy in the Δ*tsc1-11bIC* mutant. Mutant strains used for further experiments are marked with an asterisk.
(TIF)

S2 Fig Phenotype of different strains of Δ*npr1*, Δ*rhe2*, Δ*tsc1* and Δ*tsc2* mutants and the *T*. *atroviride* wild type after 10 days of growth on PDA at 25°C. (TIF)

S3 Fig Phenotype of different strains of Δ*npr1*, Δ*rhe2*, Δ*tsc1* and Δ*tsc2* mutants and the *T*. *atroviride* wild type on different nitrogen sources. Fungi were grown on minimal medium amended with selected nitrogen sources (10 ammonium sulfate, 10 mM L-glutamine, 10 mM sodium nitrate, 10 mM L-proline, or 10 mM urea) at 25°C for three days.
(TIF)

S4 Fig Joining cluster analysis from different time points (24, 48, 72 hours) of growth of *T*. *atroviride* wild type on Biolog PM3b nitrogen MicroPlates for evaluating the nitrogen source utilization capacity of the fungus. (TIF)

S5 Fig Plate confrontation assays for assessing the mycoparasitic activity of *T*. *atroviride* Δ*npr1*, Δ*rhe2*, Δ*tsc1* and Δ*tsc2* mutants and the wild type against *R*. *solani*. Fungi were inoculated on opposite sides of an agar plate containing minimal medium with 50 mM ammonium sulfate (left side: *T*. *atroviride*; right side: *R*. *solani*) and grown at 25°C for 7 days. Pictures were taken after three and seven days.
(TIF)

S6 Fig 2D-plot (m/z versus retention time) of MetExtract-derived metabolic features. Red: all metabolite ions detected in any of the four tested fungal strains. Blue: metabolite ions that carry at least one nitrogen atom in their molecular structure (nitrogen origination from NH4+ adducts was not considered).
(TIF)

S7 Fig Western blot analysis of ribosomal protein S6 (RPS6) phosphorylation in *T*. *atroviride* wild type and mutants Δ*npr1*, Δ*tsc1*, Δ*tsc2*, and Δ*rhe2* after treatment with 20 μg/ml rapamycin (+) or DMSO (-; mock control) for two hours. Samples were probed for P-RPS6, total RPS6, and histone H3 as loading control using antibodies anti-phospho-(Ser/Thr) Akt substrate, anti-Rps6, and anti-H3.
(TIF)

S8 Fig Global transcriptomic response to TOR kinase inhibition. **(A)** Scheme of the four computed comparisons for transcriptome analyses. **(B)** Heatmap of DEGs emerging from the four comparisons. Hierarchical clustering led to the identification of two main subclusters with comparisons 1 and 3 and comparisons 2 and 4 clustering together under the conditions tested.
(TIF)

## References

[1] Karlsson M, Atanasova L, Jensen DF, Zeilinger S. Necrotrophic Mycoparasites and Their Genomes. Microbiol Spectr. 2017;5(2). doi: 10.1128/microbiolspec.FUNK-0016-2016 28281442 PMC11687461

[2] Zeilinger S, Omann M. Trichoderma biocontrol: signal transduction pathways involved in host sensing and mycoparasitism. Gene Regul Syst Bio. 2007;1:227–34. doi: 10.4137/grsb.s397 19936091 PMC2759141

[3] Crespo JL, Hall MN. Elucidating TOR signaling and rapamycin action: lessons from Saccharomyces cerevisiae. Microbiol Mol Biol Rev. 2002;66(4):579–91, table of contents. doi: 10.1128/MMBR.66.4.579-591.2002 12456783 PMC134654

[4] De Craene JO, Soetens O, Andre B. The Npr1 kinase controls biosynthetic and endocytic sorting of the yeast Gap1 permease. J Biol Chem. 2001;276(47):43939–48. doi: 10.1074/jbc.M102944200 11500493

[5] Boeckstaens M, Andre B, Marini AM. The yeast ammonium transport protein Mep2 and its positive regulator, the Npr1 kinase, play an important role in normal and pseudohyphal growth on various nitrogen media through retrieval of excreted ammonium. Mol Microbiol. 2007;64(2):534–46. doi: 10.1111/j.1365-2958.2007.05681.x 17493133

[6] Boeckstaens M, Llinares E, Van Vooren P, Marini AM. The TORC1 effector kinase Npr1 fine tunes the inherent activity of the Mep2 ammonium transport protein. Nat Commun. 2014;5:3101. doi: 10.1038/ncomms4101 24476960

[7] Boeckstaens M, Merhi A, Llinares E, Van Vooren P, Springael JY, Wintjens R, et al. Identification of a Novel Regulatory Mechanism of Nutrient Transport Controlled by TORC1-Npr1-Amu1/Par32. PLoS Genet. 2015;11(7):e1005382. doi: 10.1371/journal.pgen.1005382 26172854 PMC4501750

[8] Brito AS, Soto Diaz S, Van Vooren P, Godard P, Marini AM, Boeckstaens M. Pib2-Dependent Feedback Control of the TORC1 Signaling Network by the Npr1 Kinase. iScience. 2019;20:415–33. doi: 10.1016/j.isci.2019.09.025 31622882 PMC6817644

[9] Neklesa TK, Davis RW. A genome-wide screen for regulators of TORC1 in response to amino acid starvation reveals a conserved Npr2/3 complex. PLoS Genet. 2009;5(6):e1000515. doi: 10.1371/journal.pgen.1000515 19521502 PMC2686269

[10] Schmidt A, Beck T, Koller A, Kunz J, Hall MN. The TOR nutrient signalling pathway phosphorylates NPR1 and inhibits turnover of the tryptophan permease. EMBO J. 1998;17(23):6924–31. doi: 10.1093/emboj/17.23.6924 9843498 PMC1171040

[11] Loewith R, Jacinto E, Wullschleger S, Lorberg A, Crespo JL, Bonenfant D, et al. Two TOR complexes, only one of which is rapamycin sensitive, have distinct roles in cell growth control. Mol Cell. 2002;10(3):457–68. doi: 10.1016/s1097-2765(02)00636-6 12408816

[12] Tee AR, Blenis J. mTOR, translational control and human disease. Semin Cell Dev Biol. 2005;16(1):29–37. doi: 10.1016/j.semcdb.2004.11.005 15659337

[13] Beck T, Schmidt A, Hall MN. Starvation induces vacuolar targeting and degradation of the tryptophan permease in yeast. J Cell Biol. 1999;146(6):1227–38. doi: 10.1083/jcb.146.6.1227 10491387 PMC2156124

[14] Di Como CJ, Arndt KT. Nutrients, via the Tor proteins, stimulate the association of Tap42 with type 2A phosphatases. Genes Dev. 1996;10(15):1904–16. doi: 10.1101/gad.10.15.1904 8756348

[15] Hughes Hallett JE, Luo X, Capaldi AP. State transitions in the TORC1 signaling pathway and information processing in Saccharomyces cerevisiae. Genetics. 2014;198(2):773–86. doi: 10.1534/genetics.114.168369 25085507 PMC4196627

[16] Prouteau M, Desfosses A, Sieben C, Bourgoint C, Lydia Mozaffari N, Demurtas D, et al. TORC1 organized in inhibited domains (TOROIDs) regulate TORC1 activity. Nature. 2017;550(7675):265–9. doi: 10.1038/nature24021 28976958 PMC5640987

[17] Urban J, Soulard A, Huber A, Lippman S, Mukhopadhyay D, Deloche O, et al. Sch9 is a major target of TORC1 in Saccharomyces cerevisiae. Mol Cell. 2007;26(5):663–74. doi: 10.1016/j.molcel.2007.04.020 17560372

[18] Cybulski N, Hall MN. TOR complex 2: a signaling pathway of its own. Trends Biochem Sci. 2009;34(12):620–7. doi: 10.1016/j.tibs.2009.09.004 19875293

[19] Wullschleger S, Loewith R, Hall MN. TOR signaling in growth and metabolism. Cell. 2006;124(3):471–84. doi: 10.1016/j.cell.2006.01.016 16469695

[20] Thoreen CC, Kang SA, Chang JW, Liu Q, Zhang J, Gao Y, et al. An ATP-competitive mammalian target of rapamycin inhibitor reveals rapamycin-resistant functions of mTORC1. J Biol Chem. 2009;284(12):8023–32. doi: 10.1074/jbc.M900301200 19150980 PMC2658096

[21] Atkin J, Halova L, Ferguson J, Hitchin JR, Lichawska-Cieslar A, Jordan AM, et al. Torin1-mediated TOR kinase inhibition reduces Wee1 levels and advances mitotic commitment in fission yeast and HeLa cells. J Cell Sci. 2014;127(Pt 6):1346–56. doi: 10.1242/jcs.146373 24424027 PMC3953821

[22] Serfontein J, Nisbet RE, Howe CJ, de Vries PJ. Evolution of the TSC1/TSC2-TOR signaling pathway. Sci Signal. 2010;3(128):ra49. doi: 10.1126/scisignal.2000803 20587805

[23] Inoki K, Li Y, Xu T, Guan KL. Rheb GTPase is a direct target of TSC2 GAP activity and regulates mTOR signaling. Gene Dev. 2003;17(15):1829–34. doi: 10.1101/gad.1110003 12869586 PMC196227

[24] Matsumoto S, Bandyopadhyay A, Kwiatkowski DJ, Maitra U, Matsumoto T. Role of the Tsc1-Tsc2 complex in signaling and transport across the cell membrane in the fission yeast Schizosaccharomyces pombe. Genetics. 2002;161(3):1053–63. doi: 10.1093/genetics/161.3.1053 12136010 PMC1462175

[25] van Slegtenhorst M, Carr E, Stoyanova R, Kruger WD, Henske EP. Tsc1+ and tsc2+ regulate arginine uptake and metabolism in Schizosaccharomyces pombe. J Biol Chem. 2004;279(13):12706–13. doi: 10.1074/jbc.M313874200 14718525

[26] Urano J, Tabancay AP, Yang W, Tamanoi F. The Saccharomyces cerevisiae Rheb G-protein is involved in regulating canavanine resistance and arginine uptake. J Biol Chem. 2000;275(15):11198–206. doi: 10.1074/jbc.275.15.11198 10753927

[27] Tatebe H, Shiozaki K. Evolutionary Conservation of the Components in the TOR Signaling Pathways. Biomolecules. 2017;7(4). doi: 10.3390/biom7040077 29104218 PMC5745459

[28] Panepinto JC, Oliver BG, Fortwendel JR, Smith DL, Askew DS, Rhodes JC. Deletion of the Aspergillus fumigatus gene encoding the Ras-related protein RhbA reduces virulence in a model of Invasive pulmonary aspergillosis. Infect Immun. 2003;71(5):2819–26. doi: 10.1128/IAI.71.5.2819-2826.2003 12704156 PMC153280

[29] Panepinto JC, Oliver BG, Amlung TW, Askew DS, Rhodes JC. Expression of the Aspergillus fumigatus rheb homologue, rhbA, is induced by nitrogen starvation. Fungal Genet Biol. 2002;36(3):207–14. doi: 10.1016/s1087-1845(02)00022-1 12135576

[30] Tudzynski B. Nitrogen regulation of fungal secondary metabolism in fungi. Front Microbiol. 2014;5:656. doi: 10.3389/fmicb.2014.00656 25506342 PMC4246892

[31] Lopez-Berges MS, Rispail N, Prados-Rosales RC, Di Pietro A. A nitrogen response pathway regulates virulence functions in Fusarium oxysporum via the protein kinase TOR and the bZIP protein MeaB. Plant Cell. 2010;22(7):2459–75. doi: 10.1105/tpc.110.075937 20639450 PMC2929112

[32] Teichert S, Wottawa M, Schonig B, Tudzynski B. Role of the Fusarium fujikuroi TOR kinase in nitrogen regulation and secondary metabolism. Eukaryot Cell. 2006;5(10):1807–19. doi: 10.1128/EC.00039-06 17031002 PMC1595341

[33] Yu F, Gu Q, Yun Y, Yin Y, Xu JR, Shim WB, et al. The TOR signaling pathway regulates vegetative development and virulence in Fusarium graminearum. New Phytol. 2014;203(1):219–32. doi: 10.1111/nph.12776 24684168

[34] Seidl V, Song LF, Lindquist E, Gruber S, Koptchinskiy A, Zeilinger S, et al. Transcriptomic response of the mycoparasitic fungus Trichoderma atroviride to the presence of a fungal prey. Bmc Genomics. 2009;10. doi: 10.1186/1471-2164-10-567 19948043 PMC2794292

[35] Atanasova L, Le Crom S, Gruber S, Coulpier F, Seidl-Seiboth V, Kubicek CP, et al. Comparative transcriptomics reveals different strategies of Trichoderma mycoparasitism. BMC Genomics. 2013;14:121. doi: 10.1186/1471-2164-14-121 23432824 PMC3599271

[36] Helliwell SB, Wagner P, Kunz J, Deuterreinhard M, Henriquez R, Hall MN. Tor1 and Tor2 Are Structurally and Functionally Similar but Not Identical Phosphatidylinositol Kinase Homologs in Yeast. Mol Biol Cell. 1994;5(1):105–18. doi: 10.1091/mbc.5.1.105 8186460 PMC301013

[37] Schmoll M, Dattenbock C, Carreras-Villasenor N, Mendoza-Mendoza A, Tisch D, Aleman MI, et al. The Genomes of Three Uneven Siblings: Footprints of the Lifestyles of Three Trichoderma Species. Microbiol Mol Biol Rev. 2016;80(1):205–327. doi: 10.1128/MMBR.00040-15 26864432 PMC4771370

[38] Shertz CA, Bastidas RJ, Li W, Heitman J, Cardenas ME. Conservation, duplication, and loss of the Tor signaling pathway in the fungal kingdom. BMC Genomics. 2010;11:510. doi: 10.1186/1471-2164-11-510 20863387 PMC2997006

[39] Heitman J, Movva NR, Hall MN. Targets for cell cycle arrest by the immunosuppressant rapamycin in yeast. Science. 1991;253(5022):905–9. doi: 10.1126/science.1715094 1715094

[40] Gaubitz C, Prouteau M, Kusmider B, Loewith R. TORC2 Structure and Function. Trends Biochem Sci. 2016;41(6):532–45. doi: 10.1016/j.tibs.2016.04.001 27161823

[41] Manning BD, Cantley LC. Rheb fills a GAP between TSC and TOR. Trends in Biochemical Sciences. 2003;28(11):573–6. doi: 10.1016/j.tibs.2003.09.003 14607085

[42] Huang J, Manning BD. The TSC1-TSC2 complex: a molecular switchboard controlling cell growth. Biochem J. 2008;412:179–90. doi: 10.1042/BJ20080281 18466115 PMC2735030

[43] Pfannmuller A, Wagner D, Sieber C, Schonig B, Boeckstaens M, Marini AM, et al. The General Amino Acid Permease FfGap1 of Fusarium fujikuroi Is Sorted to the Vacuole in a Nitrogen-Dependent, but Npr1 Kinase-Independent Manner. Plos One. 2015;10(4). 10.1371/journal.pone.0125487 PMC4409335 25909858

[44] Baldin C, Valiante V, Kruger T, Schafferer L, Haas H, Kniemeyer O, et al. Comparative proteomics of a tor inducible Aspergillus fumigatus mutant reveals involvement of the Tor kinase in iron regulation. Proteomics. 2015;15(13):2230–43. doi: 10.1002/pmic.201400584 25728394

[45] Seidl V, Druzhinina IS, Kubicek CP. A screening system for carbon sources enhancing beta-N-acetylglucosaminidase formation in Hypocrea atroviridis (Trichoderma atroviride). Microbiology (Reading). 2006;152(Pt 7):2003–12. 16804175 10.1099/mic.0.28897-0

[46] Zeilinger S, Gruber S, Bansal R, Mukherjee PK. Secondary metabolism in Trichoderma—Chemistry meets genomics. Fungal Biol Rev. 2016;30(2):74–90.

[47] Bueschl C, Kluger B, Lemmens M, Adam G, Wiesenberger G, Maschietto V, et al. A novel stable isotope labelling assisted workflow for improved untargeted LC-HRMS based metabolomics research. Metabolomics. 2014;10(4):754–69. doi: 10.1007/s11306-013-0611-0 25057268 PMC4098048

[48] Ceranic A, Doppler M, Buschl C, Parich A, Xu KK, Koutnik A, et al. Preparation of uniformly labelled C-13- and N-15-plants using customised growth chambers. Plant Methods. 2020;16(1). 10.1186/s13007-020-00590-9 PMC7137243 32280362

[49] Gonzalez A, Shimobayashi M, Eisenberg T, Merle DA, Pendl T, Hall MN, et al. TORC1 promotes phosphorylation of ribosomal protein S6 via the AGC kinase Ypk3 in Saccharomyces cerevisiae. PLoS One. 2015;10(3):e0120250. doi: 10.1371/journal.pone.0120250 25767889 PMC4359079

[50] Chowdhury T, Kohler JR. Ribosomal protein S6 phosphorylation is controlled by TOR and modulated by PKA in Candida albicans. Mol Microbiol. 2015;98(2):384–402. doi: 10.1111/mmi.13130 26173379 PMC4631378

[51] Loewith R, Hall MN. Target of rapamycin (TOR) in nutrient signaling and growth control. Genetics. 2011;189(4):1177–201. doi: 10.1534/genetics.111.133363 22174183 PMC3241408

[52] Weisman R. Target of Rapamycin (TOR) Regulates Growth in Response to Nutritional Signals. Microbiology Spectrum. 2016;4(5). doi: 10.1128/microbiolspec.FUNK-0006-2016 27763256

[53] Castro AF, Rebhun JF, Clark GJ, Quilliam LA. Rheb binds tuberous sclerosis complex 2 (TSC2) and promotes S6 kinase activation in a rapamycin- and farnesylation-dependent manner. J Biol Chem. 2003;278(35):32493–6. doi: 10.1074/jbc.C300226200 12842888

[54] Urano J, Comiso MJ, Guo L, Aspuria PJ, Deniskin R, Tabancay AP, et al. Identification of novel single amino acid changes that result in hyperactivation of the unique GTPase, Rheb, in fission yeast. Molecular Microbiology. 2005;58(4):1074–86. doi: 10.1111/j.1365-2958.2005.04877.x 16262791

[55] Grenson M. Study of the Positive Control of the General Amino-Acid Permease and Other Ammonia-Sensitive Uptake Systems by the Product of the Npr1 Gene in the Yeast Saccharomyces-Cerevisiae. Eur J Biochem. 1983;133(1):141–4. doi: 10.1111/j.1432-1033.1983.tb07439.x 6343084

[56] Vandenbol M, Jauniaux JC, Grenson M. The Saccharomyces-Cerevisiae Npr1 Gene Required for the Activity of Ammonia-Sensitive Amino-Acid Permeases Encodes a Protein-Kinase Homolog. Mol Gen Genet. 1990;222(2–3):393–9. doi: 10.1007/BF00633845 2125693

[57] Bastidas RJ, Shertz CA, Lee SC, Heitman J, Cardenas ME. Rapamycin Exerts Antifungal Activity In Vitro and In Vivo against Mucor circinelloides via FKBP12-Dependent Inhibition of Tor. Eukaryot Cell. 2012;11(3):270–81. doi: 10.1128/EC.05284-11 22210828 PMC3294450

[58] Pang AP, Wang HY, Zhang FN, Hu X, Wu FG, Zhou ZH, et al. High-dose rapamycin exerts a temporary impact on T. reesei RUT-C30 through gene trFKBP12. Biotechnology for Biofuels. 2021;14(1). 10.1186/s13068-021-01926-w PMC8004424 33771193

[59] Takahara T, Maeda T. TORC1 of fission yeast is rapamycin-sensitive. Genes Cells. 2012;17(8):698–708. doi: 10.1111/j.1365-2443.2012.01618.x 22762302

[60] Li LX, Zhu TT, Song Y, Luo XM, Feng L, Zhuo FP, et al. Functional Characterization of Target of Rapamycin Signaling in Verticillium dahliae. Frontiers in Microbiology. 2019;10. doi: 10.3389/fmicb.2019.00501 30918504 PMC6424901

[61] Harman GE, Howell CR, Viterbo A, Chet I, Lorito M. Trichoderma species—Opportunistic, avirulent plant symbionts. Nature Reviews Microbiology. 2004;2(1):43–56. doi: 10.1038/nrmicro797 15035008

[62] Moreno-Ruiz D, Fuchs A, Missbach K, Schuhmacher R, Zeilinger S. Influence of Different Light Regimes on the Mycoparasitic Activity and 6-Pentyl-alpha-pyrone Biosynthesis in Two Strains of Trichoderma atroviride. Pathogens. 2020;9(10). doi: 10.3390/pathogens9100860 33096850 PMC7589932

[63] Hay N, Sonenberg N. Upstream and downstream of mTOR. Gene Dev. 2004;18(16):1926–45. doi: 10.1101/gad.1212704 15314020

[64] Nakashima A, Sato T, Tamanoi F. Fission yeast TORC1 regulates phosphorylation of ribosomal S6 proteins in response to nutrients and its activity is inhibited by rapamycin. Journal of Cell Science. 2010;123(5):777–86. doi: 10.1242/jcs.060319 20144990 PMC2823578

[65] Yerlikaya S, Meusburger M, Kumari R, Huber A, Anrather D, Costanzo M, et al. TORC1 and TORC2 work together to regulate ribosomal protein S6 phosphorylation in Saccharomyces cerevisiae. Mol Biol Cell. 2016;27(2):397–409. doi: 10.1091/mbc.E15-08-0594 26582391 PMC4713140

[66] Tsao CC, Chen YT, Lan CY. A small G protein Rhb1 and a GTPase-activating protein Tsc2 involved in nitrogen starvation-induced morphogenesis and cell wall integrity of Candida albicans. Fungal Genetics and Biology. 2009;46(2):126–36. doi: 10.1016/j.fgb.2008.11.008 19095072

[67] Mach KE, Furge KA, Albright CF. Loss of Rhb1, a Rheb-related GTPase in fission yeast, causes growth arrest with a terminal phenotype similar to that caused by nitrogen starvation. Genetics. 2000;155(2):611–22. doi: 10.1093/genetics/155.2.611 10835385 PMC1461131

[68] Uritani M, Hidaka H, Hotta Y, Ueno M, Ushimaru T, Toda T. Fission yeast Tor2 links nitrogen signals to cell proliferation and acts downstream of the Rheb GTPase. Genes Cells. 2006;11(12):1367–79. doi: 10.1111/j.1365-2443.2006.01025.x 17121544

[69] Urano J, Sato T, Matsuo T, Otsubo Y, Yamamoto M, Tamanoi F. Point mutations in TOR confer Rheb-independent growth in fission yeast and nutrient-independent mammalian TOR signaling in mammalian cells. P Natl Acad Sci USA. 2007;104(9):3514–9. doi: 10.1073/pnas.0608510104 17360675 PMC1805553

[70] Tee AR, Manning BD, Roux PP, Cantley LC, Blenis J. Tuberous sclerosis complex gene products, tuberin and hamartin, control mTOR signaling by acting as a GTPase-activating protein complex toward Rheb. Curr Biol. 2003;13(15):1259–68. doi: 10.1016/s0960-9822(03)00506-2 12906785

[71] Chong-Kopera H, Inoki K, Li Y, Zhu TQ, Garcia-Gonzalo FR, Rosa JL, et al. TSC1 stabilizes TSC2 by inhibiting the interaction between TSC2 and the HERC1 ubiquitin ligase. J Biol Chem. 2006;281(13):8313–6. doi: 10.1074/jbc.C500451200 16464865

[72] Neuman NA, Henske EP. Non-canonical functions of the tuberous sclerosis complex-Rheb signalling axis. Embo Mol Med. 2011;3(4):189–200. doi: 10.1002/emmm.201100131 21412983 PMC3377068

[73] Saxton RA, Sabatini DM. mTOR Signaling in Growth, Metabolism, and Disease. Cell. 2017;168(6):960–76. doi: 10.1016/j.cell.2017.02.004 28283069 PMC5394987

[74] Peng T, Golub TR, Sabatini DM. The immunosuppressant rapamycin mimics a starvation-like signal distinct from amino acid and glucose deprivation. Mol Cell Biol. 2002;22(15):5575–84. doi: 10.1128/MCB.22.15.5575-5584.2002 12101249 PMC133939

[75] Hardwick JS, Kuruvilla FG, Tong JK, Shamji AF, Schreiber SL. Rapamycin-modulated transcription defines the subset of nutrient-sensitive signaling pathways directly controlled by the Tor proteins. P Natl Acad Sci USA. 1999;96(26):14866–70. doi: 10.1073/pnas.96.26.14866 10611304 PMC24739

[76] DeZwaan TM, Carroll AM, Valent B, Sweigard JA. Magnaporthe grisea Pth11p is a novel plasma membrane protein that mediates appressorium differentiation in response to inductive substrate cues. Plant Cell. 1999;11(10):2013–30. doi: 10.1105/tpc.11.10.2013 10521529 PMC144101

[77] Saikia S, Oliveira D, Hu GG, Kronstad J. Role of Ferric Reductases in Iron Acquisition and Virulence in the Fungal Pathogen Cryptococcus neoformans. Infection and Immunity. 2014;82(2):839–50. doi: 10.1128/IAI.01357-13 24478097 PMC3911385

[78] Moran-Diez ME, Carrero-Carron I, Rubio MB, Jimenez-Diaz RM, Monte E, Hermosa R. Transcriptomic Analysis of Trichoderma atroviride Overgrowing Plant-Wilting Verticillium dahliae Reveals the Role of a New M14 Metallocarboxypeptidase CPA1 in Biocontrol. Front Microbiol. 2019;10:1120. doi: 10.3389/fmicb.2019.01120 31191472 PMC6545926

[79] Brunner K, Peterbauer CK, Mach RL, Lorito M, Zeilinger S, Kubicek CP. The Nag1 N-acetylglucosaminidase of Trichoderma atroviride is essential for chitinase induction by chitin and of major relevance to biocontrol. Curr Genet. 2003;43(4):289–95. doi: 10.1007/s00294-003-0399-y 12748812

[80] Yu W, Mijiti G, Huang Y, Fan H, Wang Y, Liu Z. Functional analysis of eliciting plant response protein Epl1-Tas from Trichoderma asperellum ACCC30536. Sci Rep. 2018;8(1):7974. doi: 10.1038/s41598-018-26328-1 29789617 PMC5964103

[81] Salas-Marina MA, Isordia-Jasso MI, Islas-Osuna MA, Delgado-Sanchez P, Jimenez-Bremont JF, Rodriguez-Kessler M, et al. The Epl1 and Sm1 proteins from Trichoderma atroviride and Trichoderma virens differentially modulate systemic disease resistance against different life style pathogens in Solanum lycopersicum. Front Plant Sci. 2015;6:77. doi: 10.3389/fpls.2015.00077 25755658 PMC4337343

[82] Mandels M, Andreotti R. Problems and challenges in the cellulose to cellulase fermentation. Process Biochemistry. 1978;13:6–13.

[83] Colot HV, Park G, Turner GE, Ringelberg C, Crew CM, Litvinkova L, et al. A high-throughput gene knockout procedure for Neurospora reveals functions for multiple transcription factors. Proc Natl Acad Sci U S A. 2006;103(27):10352–7. doi: 10.1073/pnas.0601456103 16801547 PMC1482798

[84] Tisch D, Kubicek CP, Schmoll M. The phosducin-like protein PhLP1 impacts regulation of glycoside hydrolases and light response in Trichoderma reesei. Bmc Genomics. 2011;12. doi: 10.1186/1471-2164-12-613 22182583 PMC3267782

[85] Brunner K, Omann M, Pucher M, Delic M, Lehner S, Domnanich P, et al. Trichoderma G protein-coupled receptors: functional characterisation of a cAMP receptor-like protein from Trichoderma atroviride. Current Genetics. 2008;54(6):283–99. doi: 10.1007/s00294-008-0217-7 18836726 PMC2855678

[86] Atanasova L, Druzhinina IS. Review: Global nutrient profiling by Phenotype MicroArrays: a tool complementing genomic and proteomic studies in conidial fungi. J Zhejiang Univ Sci B. 2010;11(3):151–68. 20205302 10.1631/jzus.B1000007 PMC2833400

[87] Seo J, Shneiderman B. Interactively exploring hierarchical clustering results [gene identification]. Computer. 2002;35:80–6.

[88] Sauerschnig C, Doppler M, Bueschl C, Schuhmacher R. Methanol Generates Numerous Artifacts during Sample Extraction and Storage of Extracts in Metabolomics Research. Metabolites. 2018;8(1). 10.3390/metabo8010001 PMC5875991 29271872

[89] Kessner D, Chambers M, Burke R, Agusand D, Mallick P. ProteoWizard: open source software for rapid proteomics tools development. Bioinformatics. 2008;24(21):2534–6. doi: 10.1093/bioinformatics/btn323 18606607 PMC2732273

[90] Bueschl C, Kluger B, Neumann NKN, Doppler M, Maschietto V, Thallinger GG, et al. MetExtract II: A Software Suite for Stable Isotope-Assisted Untargeted Metabolomics. Anal Chem. 2017;89(17):9518–26. doi: 10.1021/acs.analchem.7b02518 28787149 PMC5588095

[91] R Core Team. R: A Language and Environment for Statistical Computing. 2014.

[92] Gietz RD, Sugino A. New yeast-Escherichia coli shuttle vectors constructed with in vitro mutagenized yeast genes lacking six-base pair restriction sites. Gene. 1988;74(2):527–34. doi: 10.1016/0378-1119(88)90185-0 3073106

[93] Gietz D, St Jean A, Woods RA, Schiestl RH. Improved method for high efficiency transformation of intact yeast cells. Nucleic Acids Res. 1992;20(6):1425. doi: 10.1093/nar/20.6.1425 1561104 PMC312198

[94] Jacobs P, Jauniaux JC, Grenson M. A cis-dominant regulatory mutation linked to the argB-argC gene cluster in Saccharomyces cerevisiae. J Mol Biol. 1980;139(4):691–704. doi: 10.1016/0022-2836(80)90055-8 6251229

[95] Langmead B, Salzberg SL. Fast gapped-read alignment with Bowtie 2. Nat Methods. 2012;9(4):357–9. doi: 10.1038/nmeth.1923 22388286 PMC3322381

[96] Love MI, Huber W, Anders S. Moderated estimation of fold change and dispersion for RNA-seq data with DESeq2. Genome Biol. 2014;15(12):550. doi: 10.1186/s13059-014-0550-8 25516281 PMC4302049

[97] Conesa A, Gotz S, Garcia-Gomez JM, Terol J, Talon M, Robles M. Blast2GO: a universal tool for annotation, visualization and analysis in functional genomics research. Bioinformatics. 2005;21(18):3674–6. doi: 10.1093/bioinformatics/bti610 16081474

[98] Supek F, Bosnjak M, Skunca N, Smuc T. REVIGO Summarizes and Visualizes Long Lists of Gene Ontology Terms. Plos One. 2011;6(7). doi: 10.1371/journal.pone.0021800 21789182 PMC3138752

