# Supplementary figures and images for: The TOR kinase pathway is relevant for nitrogen signaling and antagonism of the mycoparasite Trichoderma atroviride
Source: PLoS One. 2021 Dec 31;16(12):e0262180. doi: 10.1371/journal.pone.0262180 (PMC8719763; doi:10.1371/journal.pone.0262180)

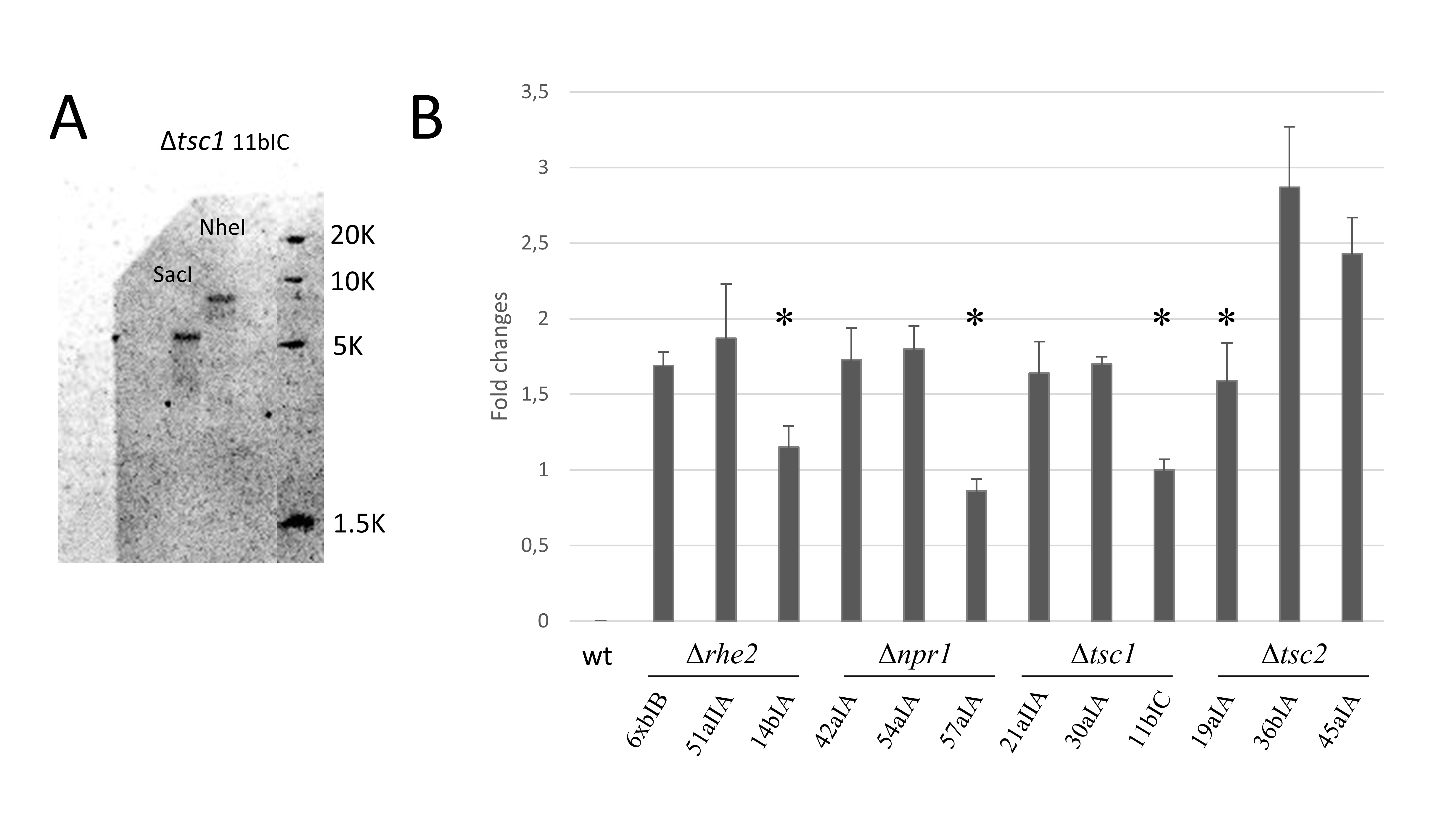

Supplement: S1 Fig — (A) Southern blot analysis to confirm single copy integration of the hph deletion cassette in the Δtsc1- 11bIC mutant. Genomic DNA was digested with SacI or NheI followed by hybridization with a DIG-labelled probe of the hph gene. (B) qPCR analysis of hph copy number in different Δrhe2, Δnpr1, Δtsc1 and Δtsc2 mutants and the wild type control. Fold changes were normalized to the single copy in the Δtsc1-11bIC mutant. Mutant strains used for further experiments are marked with an asterisk. (TIF) [file pone.0262180.s006.tif]

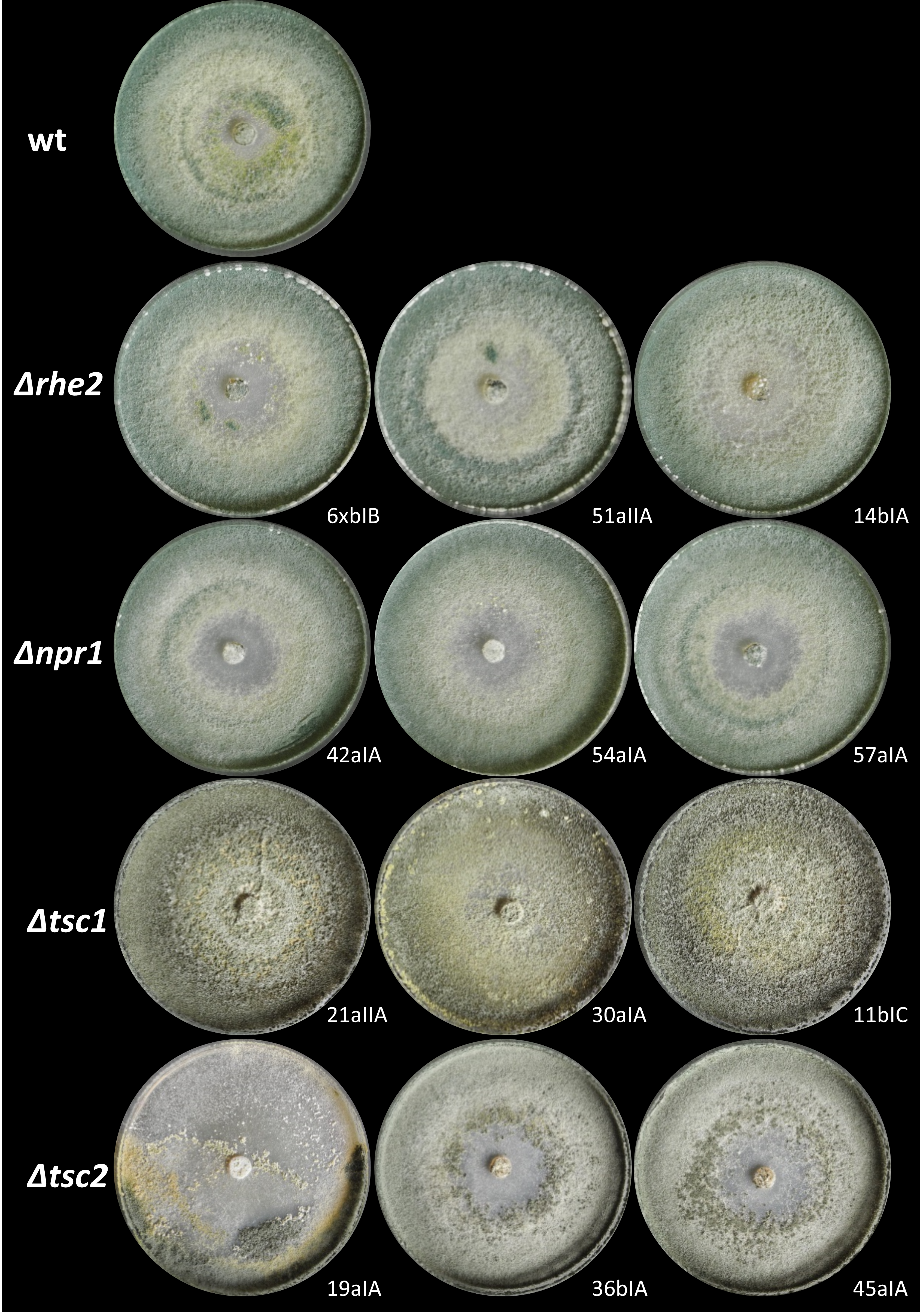

Supplement: S2 Fig — (TIF) [file pone.0262180.s007.tif]

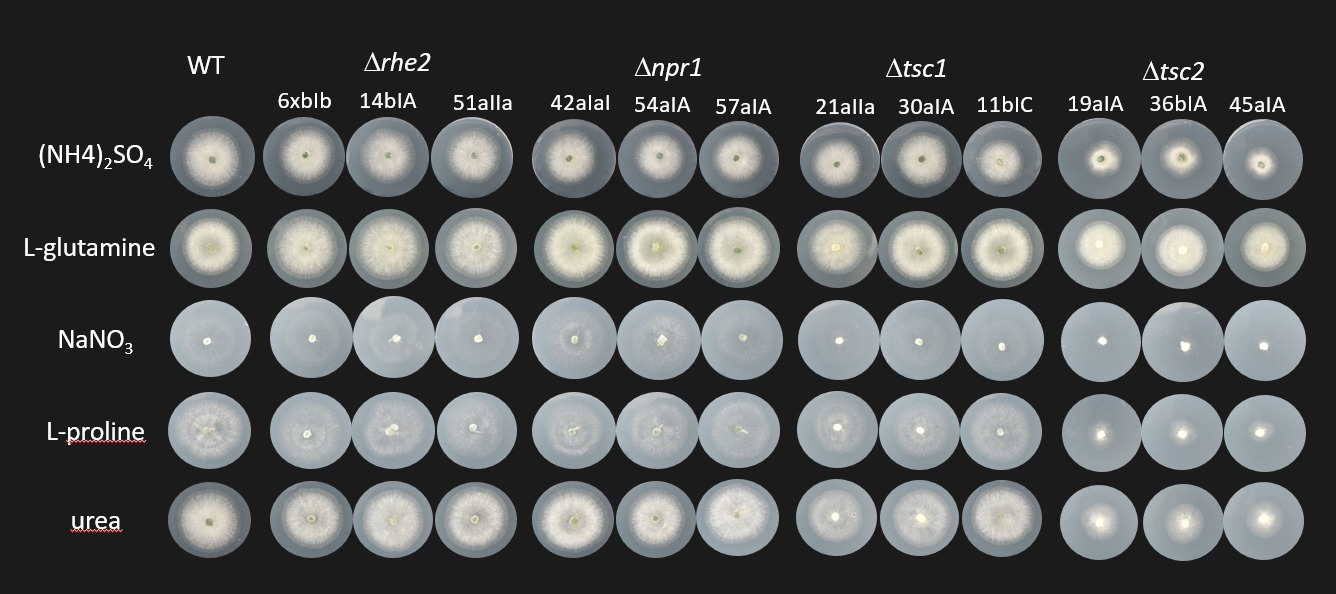

Supplement: S3 Fig — Fungi were grown on minimal medium amended with selected nitrogen sources (10 ammonium sulfate, 10 mM L-glutamine, 10 mM sodium nitrate, 10 mM L-proline, or 10 mM urea) at 25°C for three days. (TIF) [file pone.0262180.s008.tif]

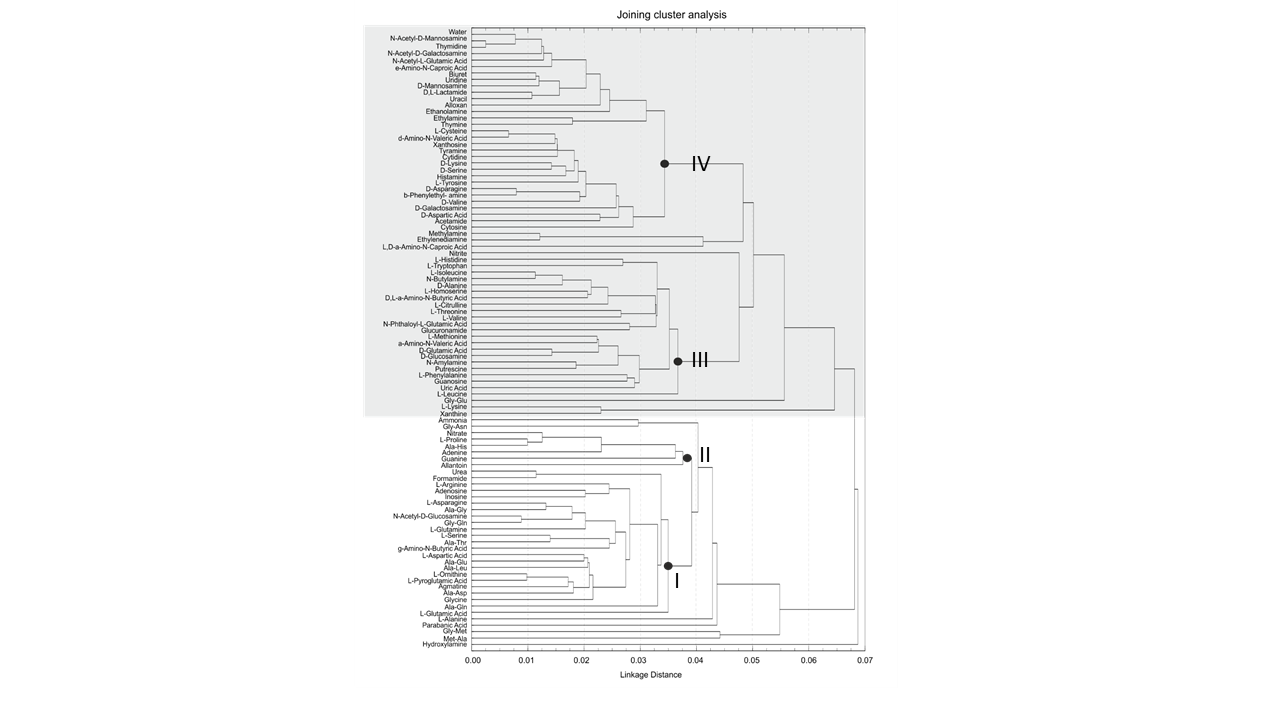

Supplement: S4 Fig — (TIF) [file pone.0262180.s009.tif]

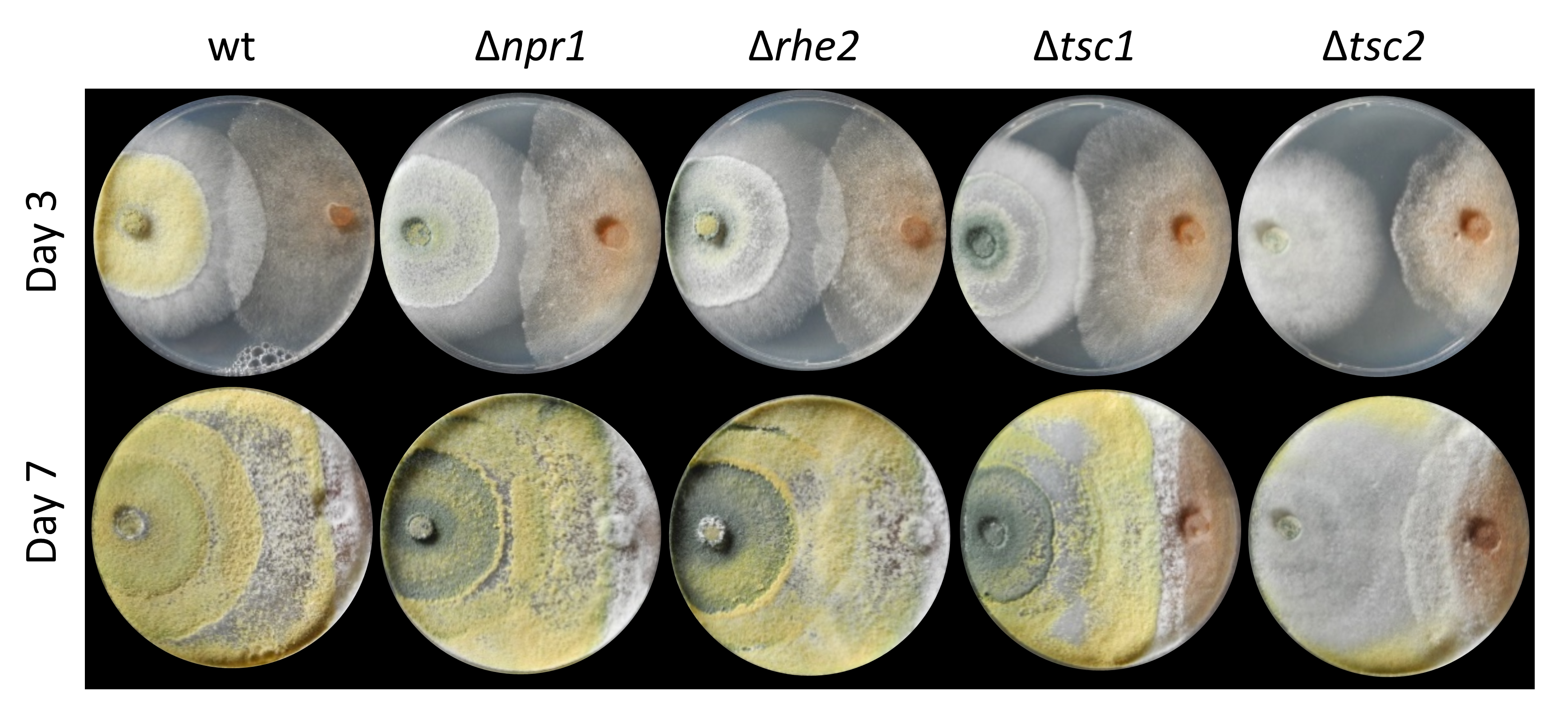

Supplement: S5 Fig — Fungi were inoculated on opposite sides of an agar plate containing minimal medium with 50 mM ammonium sulfate (left side: T. atroviride; right side: R. solani) and grown at 25°C for 7 days. Pictures were taken after three and seven days. (TIF) [file pone.0262180.s010.tif]

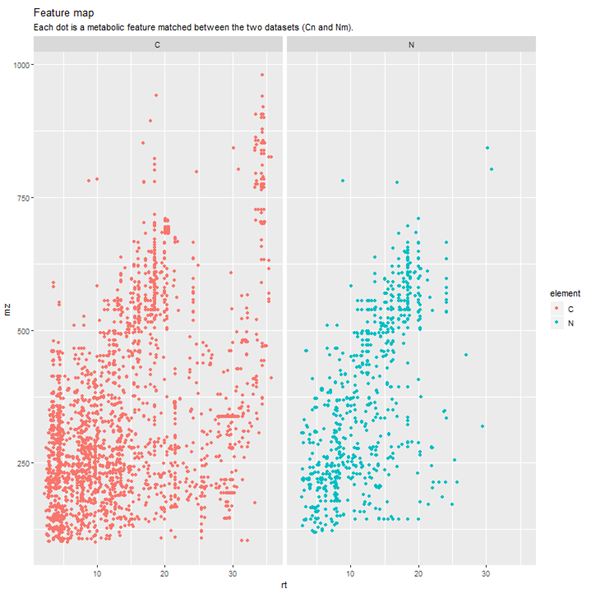

Supplement: S6 Fig — Red: all metabolite ions detected in any of the four tested fungal strains. Blue: metabolite ions that carry at least one nitrogen atom in their molecular structure (nitrogen origination from NH4+ adducts was not considered). (TIF) [file pone.0262180.s011.tif]

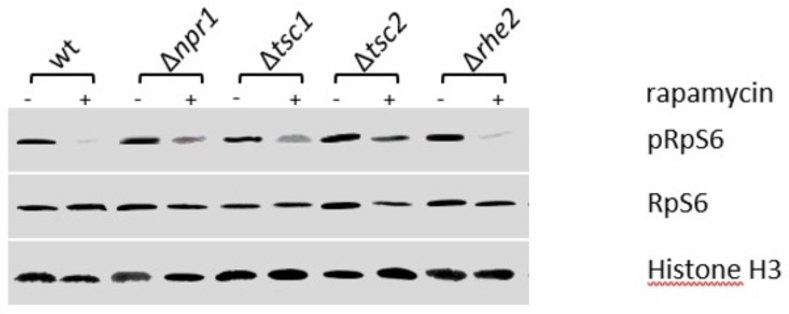

Supplement: S7 Fig — Samples were probed for P-RPS6, total RPS6, and histone H3 as loading control using antibodies anti-phospho-(Ser/Thr) Akt substrate, anti-Rps6, and anti-H3. (TIF) [file pone.0262180.s012.tif]

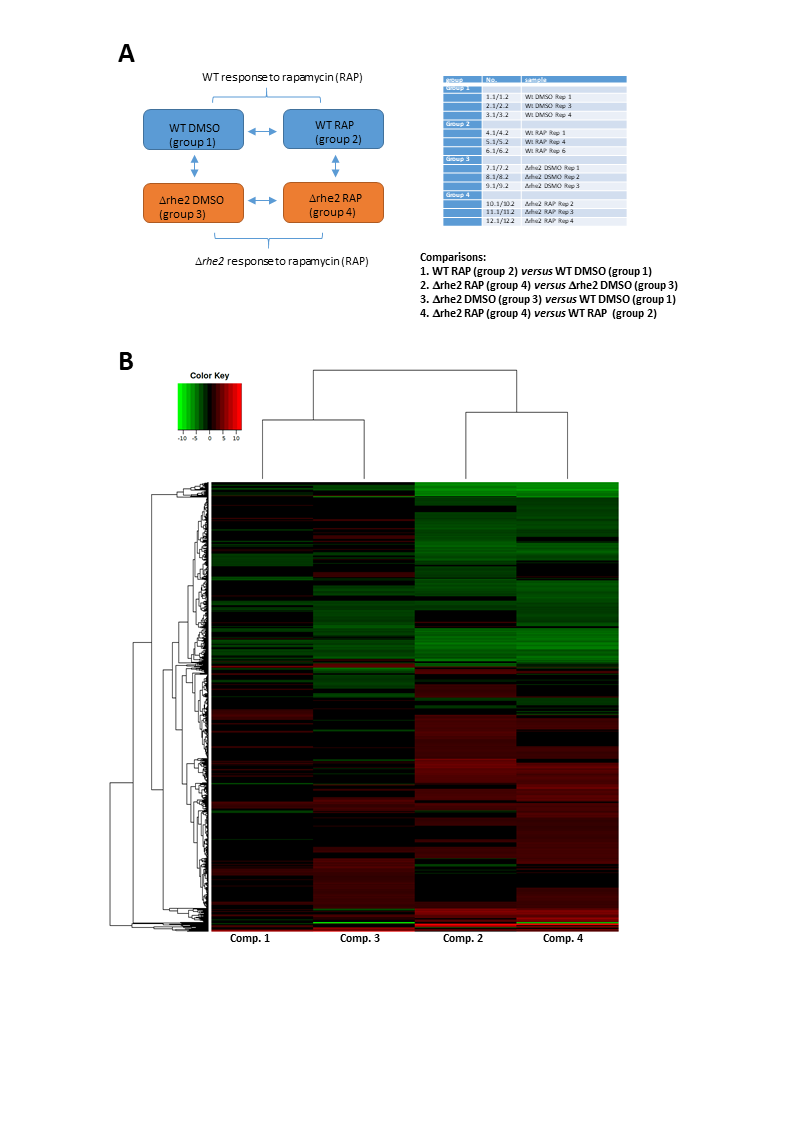

Supplement: S8 Fig — (A) Scheme of the four computed comparisons for transcriptome analyses. (B) Heatmap of DEGs emerging from the four comparisons. Hierarchical clustering led to the identification of two main subclusters with comparisons 1 and 3 and comparisons 2 and 4 clustering together under the conditions tested. (TIF) [file pone.0262180.s013.tif]
